# Autistic traits relate to speed/accuracy trade-off but not statistical learning and updating

**DOI:** 10.1038/s41598-025-16138-7

**Published:** 2025-08-30

**Authors:** Flóra Hann, Orsolya Pesthy, Bianka Brezóczki, Teodóra Vékony, Cintia Anna Nagy, Laurie-Anne Sapey-Triomphe, Eszter Tóth-Fáber, Bence Csaba Farkas, Kinga Farkas, Dezső Németh

**Affiliations:** 1 Doctoral School of Psychology, ELTE Eötvös Loránd University, Budapest, Hungary; 2 Institute of Psychology, ELTE Eötvös Loránd University, Budapest, Hungary; 3 Institute of Experimental Medicine, HUN-REN Research Centre for Natural Sciences, Budapest, Hungary; 4 Institute of Cognitive Neuroscience and Psychology, HUN-REN Research Centre for Natural Sciences, Budapest, Hungary; 5 Centre de Recherche en Neurosciences de Lyon CRNL U1028 UMR5292, INSERM, CNRS, Université Claude Bernard Lyon 1, Bron, France; 6 Gran Canaria Cognitive Research Center, Atlántico Medio University, Las Palmas de Gran Canaria, Spain; 7 Institut du Psychotraumatisme de l’Enfant et de l’Adolescent, Conseil Départemental Yvelines et Hauts-de-Seine et Centre Hospitalier des Versailles, Versailles, France; 8 Centre de Recherche en Epidémiologie et Santé des Populations, UVSQ, Inserm, Université Paris-Saclay, Versailles, France; 9 Département d’études Cognitives, LNC2, École Normale Supérieure, INSERM, PSL Research University, Paris, France; 10 Department of Psychiatry and Psychotherapy, Semmelweis University, Budapest, Hungary; 11 BML-NAP Research Group, Institute of Psychology, Eötvös Loránd University and Institute of Cognitive Neuroscience and Psychology, HUN-REN Research Centre for Natural Sciences, Budapest, Hungary

**Keywords:** ASD, Autism, Autistic traits, Predictive processing, Statistical learning, Model updating, Human behaviour, Cognitive control, Decision, Autism spectrum disorders, Learning and memory

## Abstract

Cognitive and social alterations characterize Autism Spectrum Disorder (ASD), yet comprehensive explanations are challenged by ASD’s heterogeneity. One candidate framework is predictive processing, which posits that predictive processes are altered in ASD (e.g., slower internal model updating). We tested this framework using the spectrum approach, which suggests that subclinical autistic traits are continuously distributed in the general population, with most diagnosed individuals above a threshold. We recruited neurotypical adults (*N* = 296) to examine the relationship between autistic traits and predictive processing. Using an implicit statistical learning task, we tested model updating in an unsupervised, ecologically valid manner, and assessed speed/accuracy trade-off to control for potential visuomotor performance confounds in ASD. We found no difference in model updating rate along autistic traits, suggesting no relationship between these traits and model updating in the general population, contrary to the slow updating hypothesis. However, our results reveal a difference in the evolution of speed/accuracy trade-off along the degree of autistic traits, potentially indicating a shift in the balance of goal-directed and habitual systems related to autistic traits. These findings set the stage for further research on the interaction between executive functions and predictive, habitual processes related to autistic symptoms.

**Supplementary Information:**

The online version contains supplementary material available at 10.1038/s41598-025-16138-7.

## Introduction

Difficulty with flexible adaptation to uncertainty is a characteristic of Autism Spectrum Disorder (ASD) and may be the underlying cause of certain symptoms, such as difficulties in social interactions and communication, anxiety, sensory overload, or repetitive, rigid behavior^1,2^. The essence of these adaptation difficulties is thought to lie in the alteration of predictive processing in autistic individuals, according to theories emerging in the last decade^3–5^. Predictive processing refers to the brain’s process of constructing internal models using two sources of information: prior experiences (or *priors*) and current sensory inputs^6–10^. In a world full of uncertainty, these internal models guide us based on our past experiences and help predict what comes next^11^. The theories of the predictive processing framework of ASD converge on the idea of an atypical interaction between internal predictions and sensory input and, thus, an altered anticipation of future events^2,4,5,12–15^.

Findings in this area remain inconclusive, potentially due to the heterogeneity of individuals on the autism spectrum^16–18^. The condition shows great variation in symptom manifestations as well as individual experiences^19–22^. A promising approach to capture this heterogeneity is the spectrum framework of ASD, which suggests that subclinical behavioral traits of certain conditions are continuously distributed in the general population, forming a broad phenotype, and diagnosed individuals fall above a certain threshold on this distribution^23–26^. For example, in the case of ASD, every individual in the general population can be characterized with a constellation of autistic traits (e.g., a special attention to detail and some repetitive behaviors), but often, the presence of these traits does not reach the threshold above which a diagnosis and intervention may be given^24,26–29^. However, note that an ASD diagnosis requires not only scoring high on these traits but also the co-occurrence of both social and non-social symptoms^1^.

The spectrum view in research provides multiple benefits over the binary view. First, it enables the detection of inter-individual variability in a large sample of individuals without the constraints of patient recruitment^30,31^. Large samples promote the use of fine-grained, multivariate analyses with assumptions of considerable variability^28^, enabling the detection of small but significant effects and the mitigation of noise in the data^31^. Moreover, the spectrum view helps avoid the pitfalls of case-control designs, such as certain controls being more similar to diagnosed individuals than to their own group due to having high, yet nonclinical levels of the trait of interest^32^. Recruiting non-diagnosed individuals also has the advantage of avoiding the potential masking of the effect of interest by differences in lower-level functions, such as a baseline difference in visuomotor skills in the case of statistical learning (for a similar issue in Tourette’s Syndrome, see^33^). Concerning ASD, results show alterations in visuomotor performance^34–37^, which could potentially lead to different behavioral strategies compared to non-diagnosed individuals or those with fewer autistic traits. For example, if baseline reaction time differs as a function of autistic traits, slower individuals have more room to improve than faster individuals, which could confound the interpretation of predictive abilities. To address these methodological shortcomings, firstly, we leveraged the advantages of the spectrum approach and recruited a large sample of 296 neurotypical adults. Secondly, we controlled for the possible confounding effect of a difference in visuomotor performance along autistic traits.

Prior work suggests that autistic individuals may update internal models more slowly, as shown in perceptual^14^, associative^38^, and reinforcement learning tasks^39^. However, the perceptual study allows for inferences about low-level biases but not higher-level cognitive mechanisms, while the associative and reinforcement learning studies rely on feedback or reward. This makes it difficult to isolate predictive model-building from reward-related processes – which are themselves known to be altered in ASD^40–43^. The brain is also capable of constructing internal models in an unsupervised manner, without feedback or conscious awareness^44,45^, but this domain remains unexplored in ASD. By using a task that involves complex learning without external reinforcement, our study addresses this important gap. Moreover, studies examining more complex learning often fail to capture the ambiguity and noise characteristic of real-world environments, where regularities rarely come with clear reinforcement and are seldom presented in isolation. Constructing internal models around these probabilistic regularities is known as statistical learning – a hallmark of predictive processing. In our study, we use a statistical learning task (the Alternating Serial Reaction Time task, ASRT)^45^, where participants learn a regularity by extracting it in a non-conscious way from among unpredictable elements (i.e., noise) that do not fit the pattern. This probabilistic nature of the task allows us to assess the slow updating approach with high ecological validity in the context of complex learning. Furthermore, we exploit the implicit nature of the ASRT to investigate the rate of creating and updating models in ASD in an unsupervised manner.

A version of the task used here was employed by a recent study comparing autistic and neurotypical adults^46^. They found no differences between the two groups in acquiring statistical probabilities. However, the task design did not allow for assessing model updating rate (i.e., the slow updating approach) since participants were not exposed to interfering statistical information after practicing the original one. Moreover, the small sample size typical of patient studies does not account for the diversity in populations. Thus, it did not enable the scrutiny of individual differences in predictive processing, which is crucial when investigating its associations with other constructs that may account for these differences, such as autistic traits^47^. This is also true for all of the studies described above. To address this limitation, we recruited a large sample from the general population in line with the spectrum approach, an increasingly widespread conceptualization of psychiatric conditions^23,24^. Importantly, we excluded the two participants who self-reported an ASD diagnosis from our initial sample, as our aim was to examine the association between quantified autistic traits and cognitive functioning specifically within a general population sample. We view this approach also as a valid methodological contribution to the literature, offering insight into how autistic trait variation in *neurotypical* individuals can be informative about cognitive functioning in ASD.

Using an extended version of the ASRT task used by Pesthy et al.^46^, we presented participants (without their knowledge) with two different underlying regularities in a stream of stimuli. After exposure to one implicit sequence, the sequence changed without warning, then changed back to the original one (adapting and modifying the design of Horváth et al.^48^. The incorporation of ‘old’ and ‘new’, interfering statistical information into one design allowed us to assess not just the rate at which participants acquired the ‘old’ information, but the speed at which they updated their models about the environment when exposed to interfering information. Based on Pesthy et al.^46^, we hypothesized that autistic traits would have no association with acquiring the first statistical information. Regarding internal model updating, in line with the slow updating approach^14^, we expected an association between higher autistic traits and higher resistance to interfering information during statistical learning. Therefore, we hypothesized that when exposed to a new sequence, the behavior of individuals with a higher degree of autistic traits will be governed by the already-learned information for longer compared to the behavior of those with fewer autistic traits. To control for the possible confounding effect of a difference in visuomotor performance along the spectrum of autistic traits^34–37^, we investigated the association of autistic traits and speed and accuracy separately from statistical learning. A trade-off between speed and accuracy is a typical characteristic of decision-making and learning tasks^49,50^. This trade-off measure is more complex than either speed or accuracy on its own (as analyzed by Pesthy et al.^46^), and it provides a more detailed insight into the visuomotor performance of participants. Thus, we measured the association between speed/accuracy trade-off and autistic traits, and its changes throughout the task. Based on results on visuomotor performance and ASD by Pesthy et al.^46^ and Nagy et al.^51^, we expected autistic traits to have no association with speed/accuracy trade-off.

## Methods

### Participants and screening

A total of 407 individuals participated in an online experiment. Participants were recruited through a university course from Eötvös Loránd University in Budapest and received course credits for participation. All participants gave informed consent, and the study was approved by the Research Ethics Committee of Eötvös Loránd University, Budapest, Hungary (2021/504). The authors assert that all procedures contributing to this work comply with the ethical standards, guidelines, and regulations of the relevant national and institutional committees on human experimentation and with the Helsinki Declaration of 1975, as revised in 2008.

To ensure data validity, we implemented data quality control measures. From this initial sample, we excluded participants who: (1) restarted or quit the experiment at any point (*n* = 21, 5.16%); (2) failed the attention test screening for non-compliance (*n* = 10, 2.46%); (3) had unusually low performance on the ASRT task (overall accuracy below 80%) (*n* = 13, 3.19%); (4) ​​reported neurological impairment or head injury, or reported a diagnosis of any psychiatric condition (including ASD) or epilepsy (*n* = 61, 14.99%); (5) reported having consumed alcohol within 6 hours, recreational drugs or psychoactive medications within 24 hours prior to the experiment (*n* = 36, 8.85%); (6) had a delay of more than 120 minutes between the two phases of the experiment (see below) (*n* = 1, 0.25%). As some participants met more than one exclusion criterion, these combined criteria resulted in the exclusion of a total of 111 participants (27.27%).

We designed this study to examine how quantified autistic traits relate to cognitive functioning specifically within a general population sample, which is why participants with an ASD diagnosis were excluded. Our aim was to explore cognitive variation along the spectrum of autistic traits in neurotypical individuals, offering a complementary perspective to case-control designs. Notably, only two participants in the initial sample self-reported an ASD diagnosis, so while including them might have nominally extended the continuum, their influence on overall results was expected to be minimal. Indeed, rerunning our models with these individuals included yielded no substantive changes in the findings. The interaction effects from these models are provided in Supplementary Text S1 for reference.

**Fig. 1:**
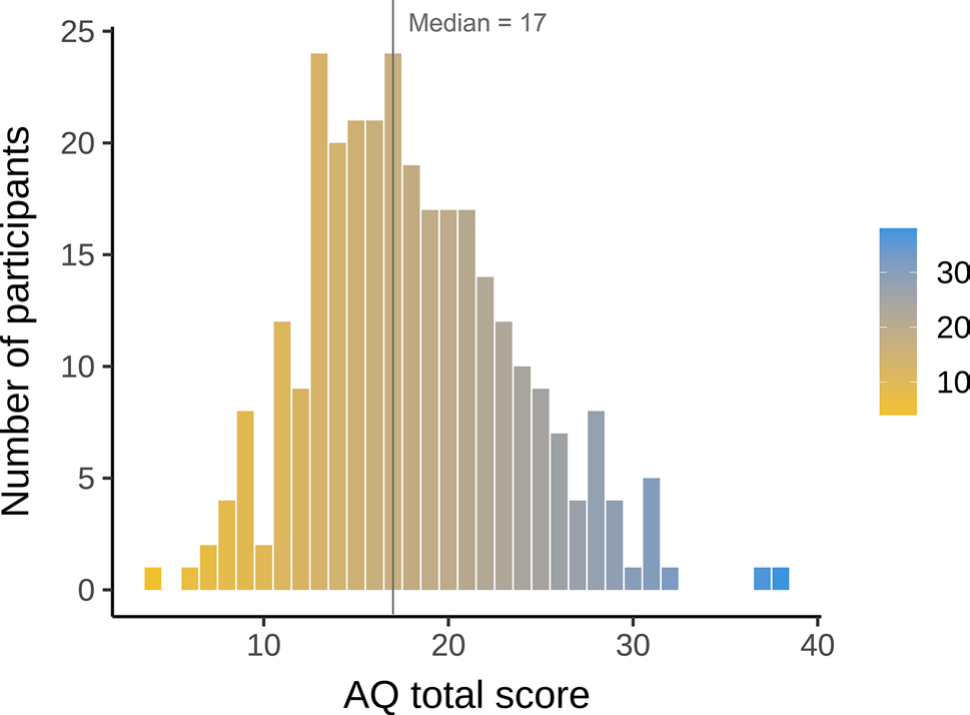
The distribution of participants’ total AQ scores (*N* = 296). Total scores represent a sum of scores on 50 self-report statements. The x-axis and the color bar indicate the total AQ score, the y-axis indicates the number of participants. The vertical line shows the median score of the sample.

The final sample after exclusion comprised 296 adult participants (213 females (72%), 79 males (27%), and 2 non-specified (1%); M_age_ = 23.2 years ± 4.98 SD, range = 18–55 years). To assess autistic traits, participants completed the Autism Spectrum Quotient (AQ)^52,^ a self-report questionnaire measuring the presence and level of autistic traits with 50 statements that have to be evaluated on a 4-point Likert scale ranging from ‘definitely disagree’ to ‘definitely agree’. Thus, higher scores indicate a higher level of autistic traits. The median score, as expected in a general population sample^53,^ was 17, with a median absolute deviation of 5.93 (Fig. 1).

### Alternating Serial Reaction Time task

**Fig. 2:**
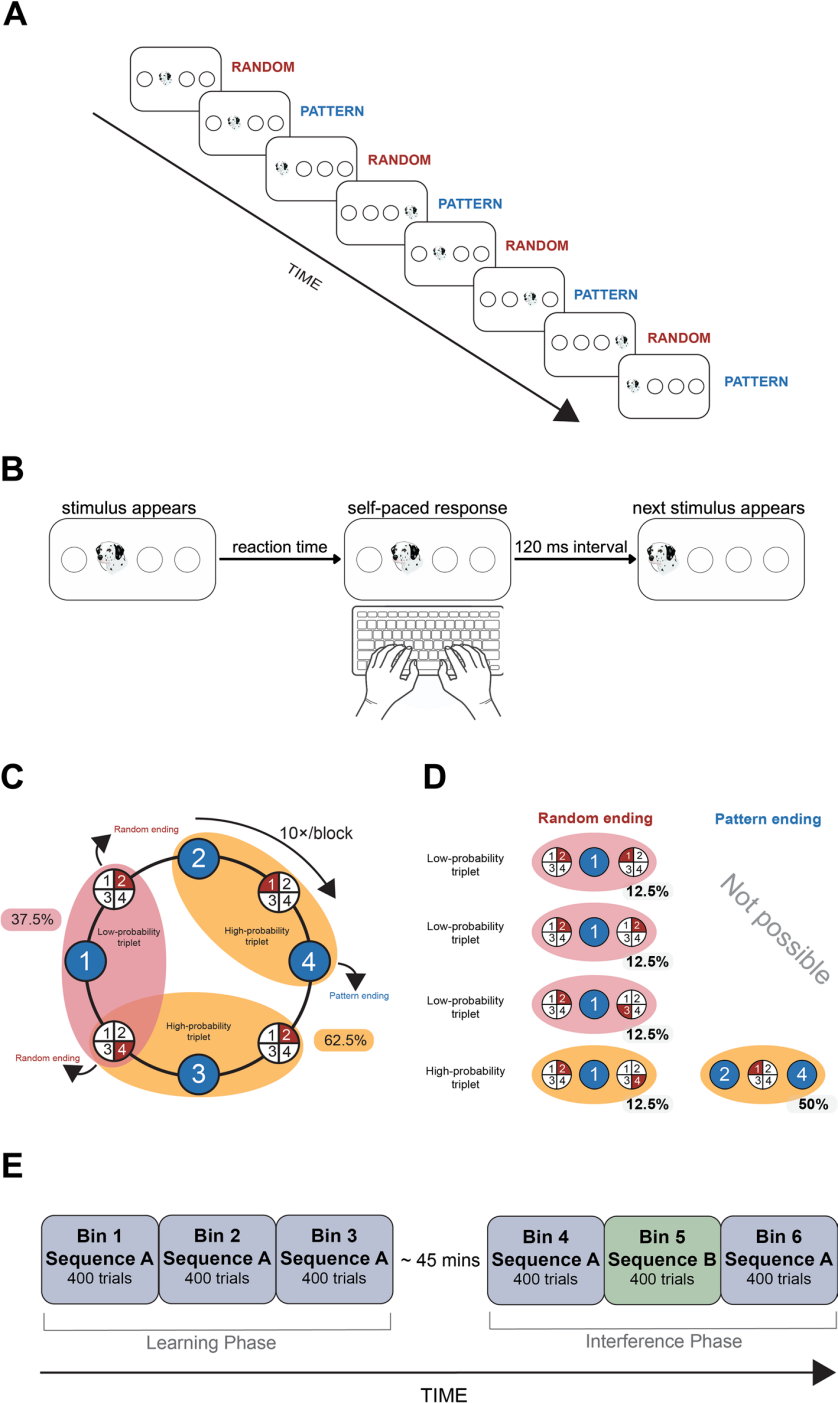
Experimental design and structure of the ASRT task. (**A**) In the ASRT task, participants had to press keys corresponding to the location of the target stimulus (dog’s head), where every second trial was part of an 8-element probabilistic sequence. Random elements were inserted among pattern elements to form the sequence (e.g., 2-r-4-r-3-r-1, where numbers indicate the location of pattern trials from left to right, and r represents random positions). (**B)** The task was self-paced, therefore stimulus presentation time was not fixed. In case of a correct response, the target stimulus disappeared, and the next stimulus appeared after a 120 ms response-stimulus interval. In case of an incorrect response, the target stimulus remained on the screen until the first correct response. (**C)** Formation of triplets in the task. Pattern elements are represented by blue backgrounds (they appear consistently in the same position throughout the task), and random elements are represented by red backgrounds (they appear randomly in one of the four possible positions). Every trial can be identified as the third element of three consecutive trials (a triplet) in a sliding window manner. The probabilistic sequence structure results in some triplets occurring with a higher frequency (high-probability triplets, 62.5% of all trials) than others (low-probability triplets, 37.5% of all trials). Statistical learning is operationalized as the performance improvement in high-probability trials compared to low-probability trials. Ten repetitions of the 8-element sequence (80 trials) make up one block of the task. (**D)** The formation of high-probability triplets could have involved the occurrence of either two pattern trials with one random trial between them, which was the case in 50% of trials; or two random trials with one pattern trial between them, which occurred in 12.5% of trials. In total, 62.5% of all trials constituted the final element of a high-probability triplet, while the remaining 37.5% were the final elements of a low-probability triplet. (**E)** The experiment consisted of a learning phase, where participants were exposed to the same sequence (Sequence A) for a total of 1200 trials (divided into 3 bins of 400 trials each for analyses). The learning phase was followed by a delay of 45 min on average, where participants filled out questionnaires. Then, in the interference phase, participants first practiced Sequence A again in Bin 4. In Bin 5, the underlying sequence was changed without warning to the reversed version of the original sequence (Sequence B). Lastly, in Bin 6, participants were again presented with Sequence A.

To measure statistical learning and visuomotor performance, we used the ASRT task^45^. The task was programmed in JavaScript using the jsPsych library v.6.1.0^54,55^. In each trial, participants were presented with a visual target stimulus (a drawing of a dog’s head) in one of four horizontal circles on the screen (Fig. 2A). Participants were instructed to indicate the location of the target stimulus by pressing the corresponding key on the keyboard (S, F, J, or L keys from left to right) as quickly and accurately as possible. The specific instructions were as follows: ‘*In this task*,* you will see four circles in the center of the screen. The keys ‘S’*,* ‘F’*,* ‘J’*,* and ‘L’ correspond to the circles from left to right. From time to time*,* a dog’s head will appear in one of the circles. Your task will be to ‘catch the dog’ by pressing the corresponding key as accurately and fast as possible. During the task*,* there will be short breaks every few minutes*,* during which you will get feedback on your average accuracy and reaction time*,* which you can use to improve your performance’*. The task was self-paced, therefore, stimulus presentation time was not fixed. In case of a correct response, the target stimulus disappeared, and the next stimulus appeared after a 120 ms response-stimulus interval. In case of an incorrect response, the target stimulus remained on the screen until the first correct response. After each short break, the first stimulus of the next block appeared after a 1000 ms interval (Fig. 2B). Unbeknownst to the participants, the appearance of the stimuli followed a probabilistic eight-element sequence with pattern and random elements alternating with each other (Fig. 2A, e.g., 2-r-4-r-3-r-1-r, where r indicates a random location, and the numbers represent the predetermined positions (circles) from left to right). Each participant was randomly assigned one of six possible sequences.

The task features a probabilistic structure, where runs of three consecutive stimuli (triplets) could be categorized into high- and low-probability triplets based on how likely they were to appear in that specific order. A trial refers to a single element in the sequence that could be either a pattern or a random element and, crucially, also to the last element in a high- or low- probability triplet. For example, in a sequence such as 2-r-4-r-3-r-1-r, triplets such as 2-X-4, 4-X-3, 3-X-1, and 1-X-2 (where X represents the middle element of a triplet, which could be either pattern or random) occurred more frequently (i.e., with higher probability) than triplets such as 2-X-1 or 2-X-3, as their first and third elements could be either a pattern or a random trial (Fig. 2C). When referring to ‘triplet type’ throughout the rest of this paper, the focus is on trials serving as the last element of a high- or low-probability triplet.

Throughout the task, a total of 64 distinct triplets could occur: 16 with high probability and 48 with low probability. High-probability triplets could be formed by either two pattern trials with one random trial between them (occurring in 50% of trials) or by two random trials with one pattern trial between them (occurring in 12.5% of trials). Of all trials, 62.5% represented the last element of these high-probability triplets, and 37.5% represented the last element of low-probability triplets (Fig. 2D). Robust evidence demonstrates performance facilitation over time in both reaction time (RT) and accuracy to high-probability triplets compared to low-probability triplets. This indicates that participants become sensitive to the underlying probabilistic regularity in the task^45,56–60^. Moreover, this knowledge is entirely implicit, with no evidence of explicit awareness as evaluated through self-reports and sequence generation tasks^61^. We also confirmed this by asking our participants after the ASRT task whether they noticed anything unusual, or any regularities in the task, and if so, they were asked to elaborate. None of the participants could give an accurate description of the sequence or noticed its existence. Therefore, the performance differences between high- and low-probability triplets indicate implicit statistical learning. Additionally, overall performance in RT and accuracy improves during the task regardless of triplet-probability, indicating visuomotor performance improvement. The ability to distinguish between these two processes is a key strength of the ASRT task (e.g^62^.,).

### Procedure

The online experiment was hosted on Gorilla Experiment Builder (https://gorilla.sc)^63^. The ASRT task was divided into blocks of 80 trials: each block comprised 10 repetitions of the 8-element sequence, with short (< 1 min), self-paced rest periods between blocks. Here, participants received feedback on their RT and accuracy (but not on their learning performance, as they were unaware that the task involved learning). First, participants completed two practice blocks with no underlying sequence (i.e., only random trials). These two blocks served only to familiarize participants with the task and were not analyzed. To facilitate data analysis, we aggregated the remaining blocks into units of 5 blocks (or 400 trials), hereafter referred to as bins. The experiment was divided into a learning phase (Bins 1–3) and an interference phase (Bins 4–6), with a self-paced delay period between them (Fig. 2E), where participants filled out the AQ^52^ among other questionnaires not analyzed in this study. This rest period lasted 44.6 min on average (± 19.4 SD).

The learning phase comprised 3 bins of the ASRT task (15 blocks or 1200 trials), with an underlying sequence (Sequence A, Fig. 2E). In this phase, we can measure the acquisition of the probabilistic regularity by comparing performance on high- and low-probability triplets and the overall effect of practice in visuomotor performance by comparing performance between successive bins. Then, in the interference phase, participants first practiced Sequence A again in Bin 4. In Bin 5, the underlying sequence was changed without warning to the reversed version of the original sequence (Fig. 2E), e.g., if Sequence A is 2-r-4-r-3-r-1-r, then Sequence B is 1-r-3-r-4-r-2-r. Lastly, in Bin 6, participants are again presented with Sequence A (without warning). This interference phase allows for comparing performance on Sequence B following Sequence A, then again on Sequence A following Sequence B. This way, we can measure how a well-learned environmental regularity affects the acquisition of a novel regularity and how that interfering novel regularity disrupts the established implicit knowledge of the previously learned one.

### Statistical analyses

#### Preprocessing

Responses in the ASRT task were processed using R (version 4.3.3)^64^. Each trial was categorized as the last element of a high- or low-probability triplet based on the two preceding trials. Trills (e.g., 1-2-1; 9.14%) and repetitions (e.g., 2-2-2; 3.06%) were removed, as participants can show pre-existing response tendencies to these trial types, such as automatic facilitation^65^. We also removed the first two trials of each block (2.50%), as they do not form a third element of a triplet, so their probability category cannot be identified. Trials with RT > 1000 ms (0.36%) were also removed to ensure data validity. Finally, incorrect responses were removed from the analysis of RTs (9.18%). Please note that these criteria overlapped in the case of some trials. Based on these criteria, 22.43% of trials were removed in total for the final analysis of RTs (per participant: M = 0.08% of trials ± 0.01% SD, range = 0.05–0.10%) and 15.97% of trials for the final analysis of accuracy (per participant: M = 0.05% of trials ± < 0.01% SD, range = 0.05–0.61%).

#### Statistical learning scores

Statistical learning (or the extraction of regularities from the environment) was operationalized as the difference in binwise median RT and mean accuracy between high- and low-probability trials (i.e., between the third element of a high-probability triplet and the third element of a low-probability triplet), hereafter referred to as statistical learning score. Higher scores indicate better learning. We also calculated inverse efficiency scores (IES) by dividing binwise median RT by mean accuracy. Then, we calculated IES learning scores by subtracting the IES of high-probability triplets from the IES of low-probability triplets, as higher IES indicates worse performance, i.e., slower and/or less accurate responses.

#### Speed/accuracy trade-off scores

To maximize performance, a trade-off between speed and accuracy is a typical characteristic of decision-making and learning tasks^49,50^. Even though this phenomenon is general in cognitive tasks, literature on the presence and dynamics of this trade-off in statistical learning is scarce. Given that RT and accuracy changed oppositely in the task, we explored their trade-off, obtaining a more complex measure than either speed or accuracy on its own.

To quantify speed/accuracy trade-off (SAT) for each participant in each bin, we calculated a variable hereafter referred to as trade-off score, representing the relative bias toward faster or more accurate responses, by subtracting a within-participant z-scored error rate from a within-participant z-scored RT score in the given bin (following Vékony et al.^66)^. Thus, values close to zero indicate a relatively bias-free response style, more positive values indicate a more accurate response style at the expense of speed (i.e., a participant’s responses in that particular bin were more accurate than their participant-specific mean and/or slower than their participant-specific mean RT), while negative values indicate a faster response style at the expense of accuracy (i.e., a participant’s responses in that particular bin were less accurate than their participant-specific mean and/or faster than their participant-specific mean RT).

#### Statistical modeling

Data analysis was performed using R (version 4.3.3)^64^. Linear mixed models were fitted with the *mixed* function from the *afex* package (version 1.10.1)^67^ to test the relationship between autistic traits and statistical learning (Model_SL−RT_ and Model_SL−ACC_), model updating (Model_U−RT_ and Model_U−ACC_), and speed/accuracy trade-off (Model_SAT_ and Model_SAT−AQ_). The outcome variables were statistical learning score for Model_SL−RT_, Model_SL−ACC_, Model_U−RT_, and Model_U−ACC_, binwise median RT for Model_SAT_, and speed/accuracy trade-off score for Model_SAT−AQ_ (as described above). The predictor variables were bin (within-subject) and AQ (between-subject) scores for all models except for Model_SAT−AQ_, where the predictors were bin and binwise mean accuracy. Bin was a factor variable (Bin 1–3 for Model_SL−RT_ and Model_SL−ACC_, Bin 4–6 for Model_U−RT_ and Model_U−ACC_, and Bin 1–6 for Model_SAT_ and Model_SAT−AQ_), coded to reflect the progression of the task in time, with the first relevant bin as reference in all models. The AQ score was continuous and mean-centered. The models included the predictor variables as fixed effects, and participant-specific intercepts as random effects. Thus, the models corresponded to the following general form in *lmer* syntax:

1. Statistical learning:

 Model_SL−RT_: *RT learning score ~ Bin*_*1−3*_ *× AQ + (1 | Participant)*

 Model_SL−ACC_: *ACC learning score ~ Bin*_*1−3*_ *× AQ + (1 | Participant)*

2. Model updating:

 Model_U−RT_: *RT learning score ~ Bin*_*4−6*_ *× AQ + (1 | Participant)*

 Model_U−ACC_: *ACC learning score ~ Bin*_*4−6*_ *× AQ + (1 | Participant)*

3. Speed/accuracy trade-off:

 Model_SAT_: *Binwise median RT ~ Bin*_*1−6*_ *× Binwise mean accuracy + (1 | Participant)*

4. Speed/accuracy trade-off and AQ:

 Model_SAT−AQ_: *SAT score ~ Bin*_*1−6*_ *× AQ + (1 | Participant)*

Assumptions of linearity, normal distribution of residuals, homoscedasticity, and multicollinearity were evaluated using the *sjPlot* (version 2.8.15)^68^,* performance* (version 0.11.0)^69^, and *car* (version 3.1-3)^70^ packages in R, using scatterplots, QQ plots, and statistical tests. Assumptions of linearity and homoscedasticity were met in all cases. Recent work indicated that fixed effect estimates of linear mixed models are robust to violations of the assumption of residual normality^71,72^. Thus, we deemed our models adequate, even though this assumption was slightly violated in all cases.

Sample sizes in terms of total number of data points and of sampling units, random effects estimates, Nakagawa’s marginal and conditional R^2^s^73,^ and the adjusted Intraclass Correlation Coefficients (ICCs) are all consistently reported in the summary tables (Supplementary Tables S1-S5, S8). Post-hoc contrasts were conducted with the *emmeans* R package after regridding (version 1.10.1)^74^. We used Type III tests for inference about fixed effects. We obtained degrees of freedom using the Satterthwaite approximation^75^. An alpha level of 0.05 was applied to all analyses. *P* values of all post-hoc tests were adjusted with the Šidák method in case of multiple comparisons^76^. All *p* values are two-tailed. Figures were created with the *ggplot2* package (version 3.5.1)^77^, supplemented by the *cowplot* package (version 1.1.3)^78^. All data and codes for data analysis and visualization are available on the following OSF repository: https://osf.io/5tsgy/.

## Results

All models included AQ sum score as a continuous variable. However, for interpretability, we also visualized results using separate lines for two median-split AQ groups. Figures always plot raw data, whereas post-hoc tests are done using model-estimated marginal means. For post-hoc estimates of continuous variables, we used M ± 1 SD to represent ‘lower’ and ‘higher’ values of the given variable. We also ran models for statistical learning and model updating using inverse efficiency score learning scores as outcome variables, but their results closely mirrored those of the separate RT and accuracy models, so they are not reported here.

### Are autistic traits associated with the acquisition of statistical information?

First, we investigated the change in statistical learning performance in the task, that is, the difference in RT and accuracy performance between high- and low-probability triplets. We tested whether this change is associated with autistic traits. We computed a median RT learning score and a mean accuracy learning score (see above) for each participant for each bin in Bins 1–3. These measures were the outcome variables in Model_SL−RT_ and Model_SL−ACC_, respectively, with Bin, AQ score, and their interactions as fixed effects. We first fit the maximal random effect structures justified by the design: including a random intercept for each participant and a random slope for the interaction of Bin and AQ score. However, the models achieved convergence when only a random intercept was fit.

#### Analysis based on reaction times

**Fig. 3:**
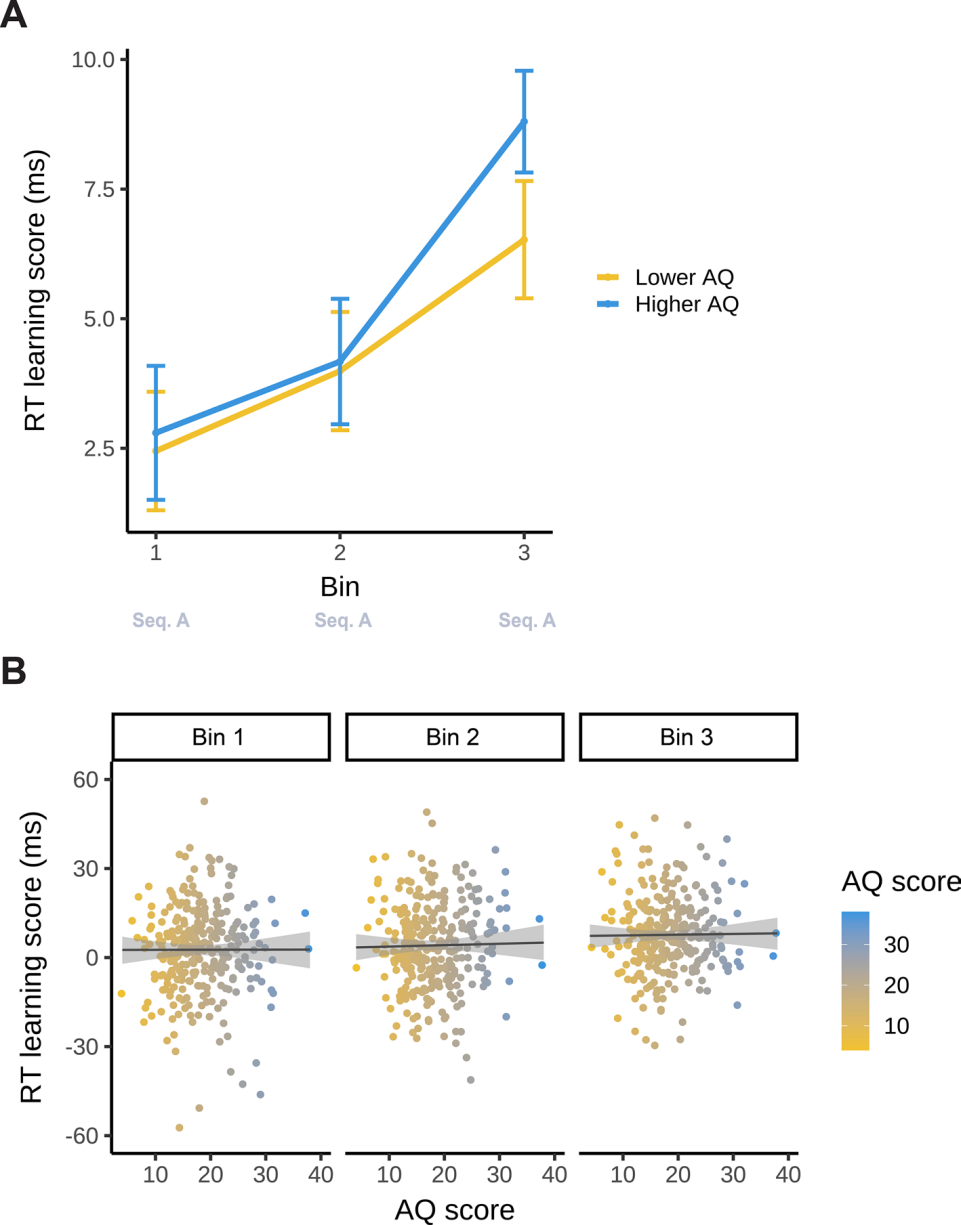
Statistical learning (reaction time) as a function of AQ. The y-axis represents performance in terms of binwise RT learning scores (the difference in binwise median RT between low- and high-probability triplets). (**A)** Statistical learning trajectories are shown in the learning phase of the task (Bin 1–3). Please note that AQ scores were entered in the analyses as continuous variables, but here, for interpretability, we also visualized AQ by median-splitting the sample into two groups. The yellow line represents lower AQ (scores 4–17), and the blue line represents higher AQ individuals (scores 18–38). The x-axis represents bins of the ASRT task (the progression of time). Error bars show 1 SEM. (**B)** Correlations between individual AQ sum scores and binwise RT learning scores are shown in the learning phase of the task (Bin 1–3). The x-axis and the color bar represent AQ sum scores. The lines show the linear trend, and the shades reflect 1 SEM.

Model_SL−RT_ reported a significant main effect of Bin (*F*(2, 588) = 10.679; *p* < 0.001). This indicates that statistical learning improved gradually during the task: there was an increasingly higher difference in RT between high- and low-probability triplets as the task progressed (Bin 1: 2.62 ms, 95% CI = [1.02, 4.22]; Bin 2: 4.08 ms, 95% CI = [2.48, 5.68]; Bin 3: 7.66 ms, 95% CI = [6.05, 9.26]; Bin 1–2 contrast *p*_*cor*_ = 0.457, Bin 1–3 contrast *p*_*cor*_ < 0.001, Bin 2–3 contrast *p*_*cor*_ = 0.004) (Fig. 3).

Neither the main effect of AQ (*F*(1, 294) = 0.081; *p* = 0.776), nor the interaction between Bin and AQ was significant (*F*(2, 588) = 0.025; *p* = 0.975). This suggests that AQ had no association with either statistical learning in general or its trajectory throughout the task in terms of RTs (Fig. 3). The full list of fixed and random effect parameters can be found in Supplementary Table S1.

#### Analysis based on accuracy

**Fig. 4:**
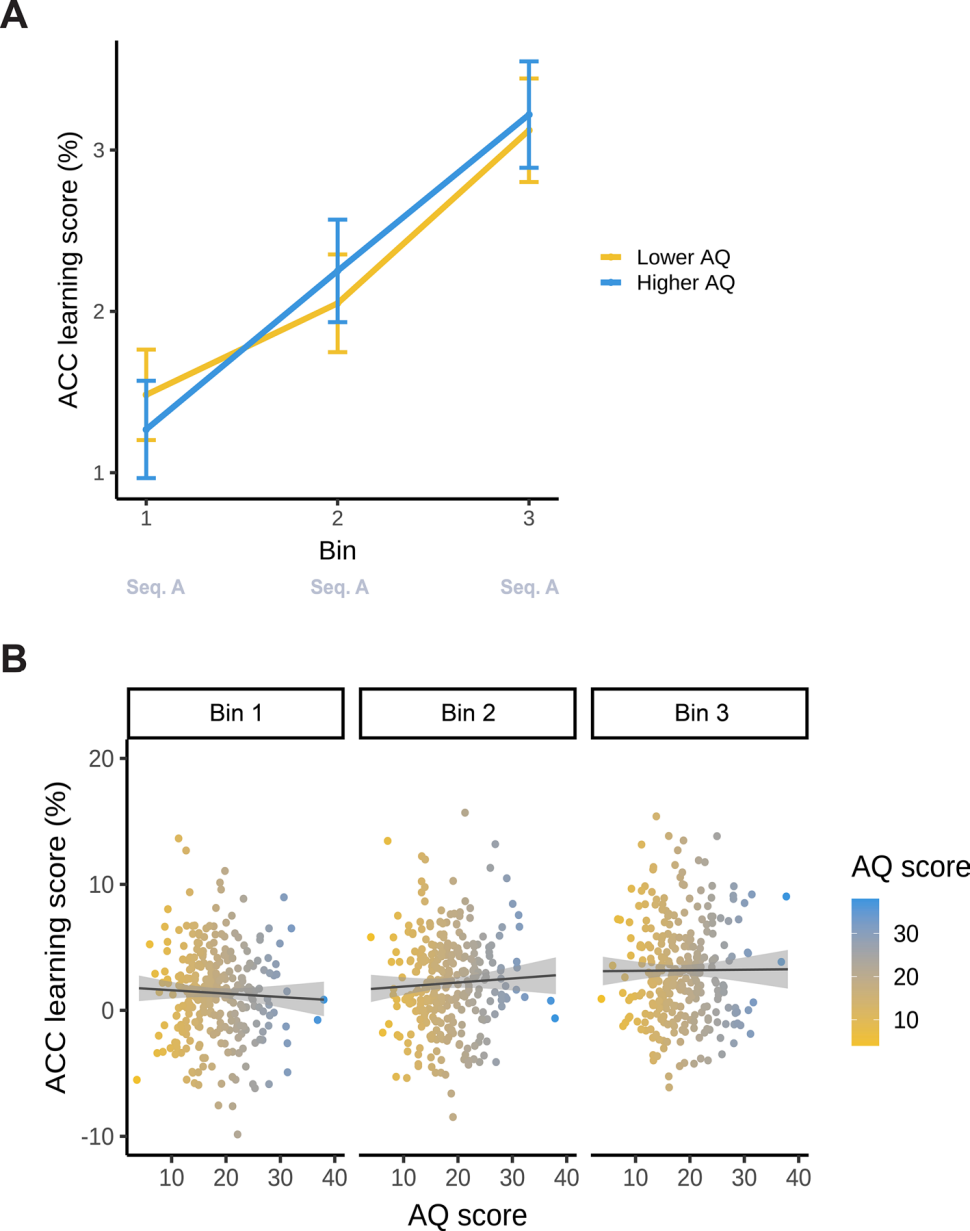
Statistical learning (accuracy) as a function of AQ. The y-axis represents performance in terms of binwise accuracy learning scores (the difference in binwise mean accuracy between high- and low-probability triplets). (**A)** Statistical learning trajectories are shown in the learning phase of the task (Bin 1–3). Please note that AQ scores were entered in the analyses as continuous variables, but here, for interpretability, we also visualized AQ by median-splitting the sample into two groups. The yellow line represents lower AQ (scores 4–17), and the blue line represents higher AQ individuals (scores 18–38). The x-axis represents bins of the ASRT task (the progression of time). Error bars show 1 SEM. (**B)** Correlations between individual AQ sum scores and binwise accuracy learning scores are shown in the learning phase of the task (Bin 1–3). The x-axis and the color bar represent AQ sum scores. The lines show the linear trend, and the shades reflect 1 SEM.

Model_SL−ACC_ resulted in a statistically significant main effect of Bin (*F*(2, 588) = 18.141; *p* < 0.001). Similarly to RT results, this indicates that statistical learning changed gradually during the task: there was an increasingly higher difference in accuracy between high- and low-probability triplets as the task progressed (Bin 1: 1.38%, 95% CI = [0.95, 1.81]; Bin 2: 2.15%, 95% CI = [1.72, 2.58]; Bin 3: 3.17, 95% CI = [2.74, 3.60]; contrasts for all pairs of bins *p*_*cor*_ ≤ 0.029) (Fig. 4).

Neither the main effect of AQ (*F*(1, 294) = 0.182; *p* = 0.893), nor the interaction between Bin and AQ was significant (*F*(2, 588) = 0.616; *p* = 0.540). This suggests that AQ had no association with either statistical learning in general or its trajectory throughout the task in terms of accuracy (Fig. 4). The full list of fixed and random effect parameters can be found in Supplementary Table S2.

### Are autistic traits associated with resistance to interfering statistical information?

Next, we examined whether the exposure to an interfering sequence in the task (Sequence B) influences performance on the already-learned information for longer, and whether autistic traits will modulate this potential change. Again, we computed a median RT learning score and a mean accuracy learning score for each participant for each bin in Bins 4–6. These measures were the outcome variables in Model_U−RT_ and Model_U−ACC_ respectively, with Bin, AQ score, and their interactions as fixed effects. We first fit the maximal random effect structures justified by the design: including a random intercept for each participant and a random slope for the interaction of Bin and AQ score. However, the models achieved convergence when only a random intercept was fit.

#### Analysis based on reaction times

**Fig. 5:**
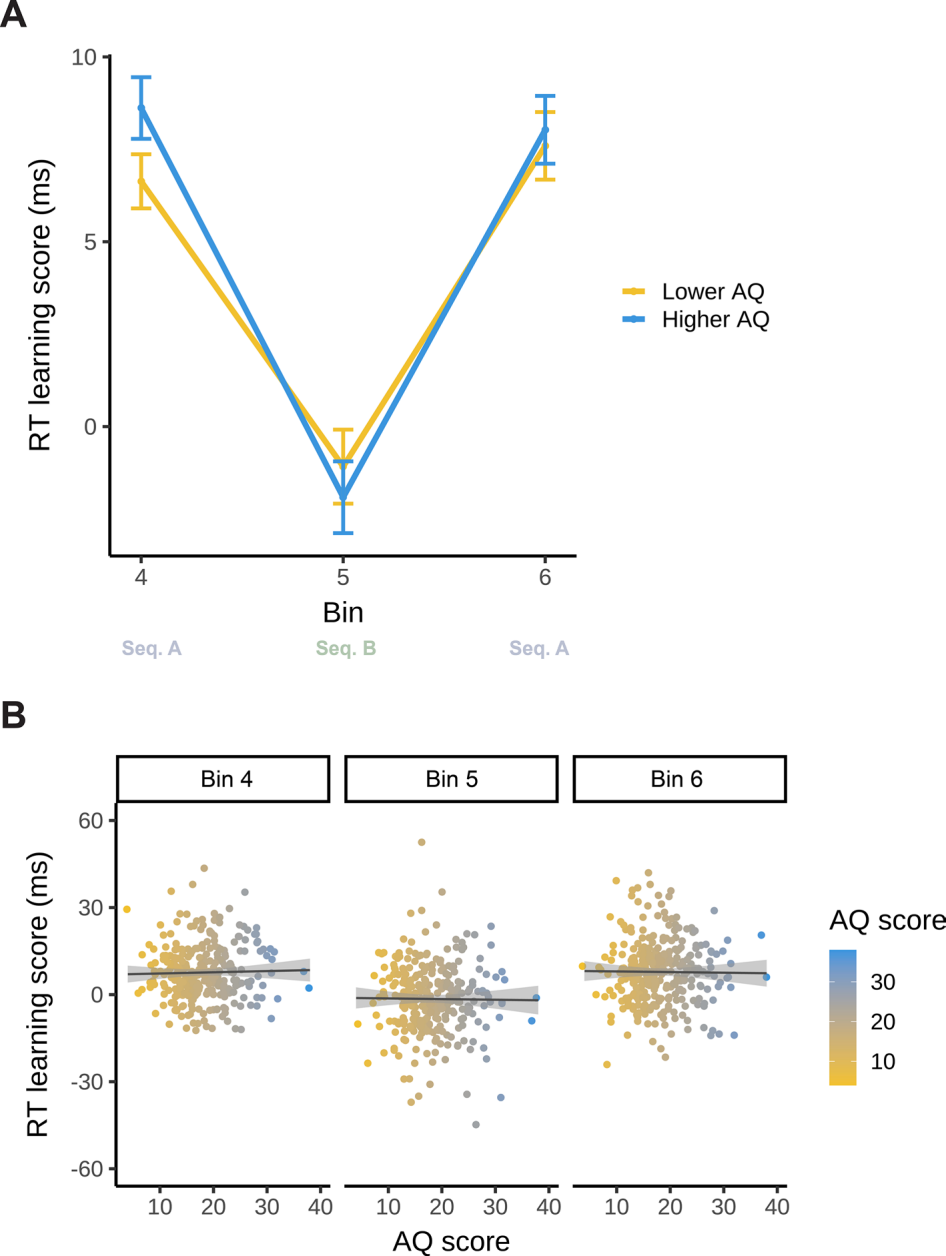
Model updating (reaction time) as a function of AQ. The y-axis represents performance in terms of binwise RT learning scores (the difference in binwise median RT between low- and high-probability triplets). (**A)** Statistical learning trajectories are shown before (Bin 4), during (Bin 5), and after (Bin 6) exposure to interfering statistical information. Please note that AQ scores were entered in the analyses as continuous variables, but here, for interpretability, we also visualized AQ by median-splitting the sample into two groups. The yellow line represents lower AQ (scores 4–17), and the blue line represents higher AQ individuals (scores 18–38). The x-axis represents bins of the ASRT task (the progression of time). Error bars show 1 SEM. (**B)** Correlations between individual AQ sum scores and binwise RT learning scores are shown before (Bin 4), during (Bin 5), and after (Bin 6) exposure to interfering statistical information. The x-axis and the color bar represent AQ sum scores. The lines show the linear trend, and the shades reflect 1 SEM.

Model_U−RT_ resulted in a statistically significant main effect of Bin (*F*(2, 822) = 69.713; *p* < 0.001). This indicates that statistical learning changed gradually during the task according to sequence identity (A or B): the difference in RT between high- and low-probability triplets decreased between Bin 4 (Sequence A) and Bin 5 (Sequence B), then increased again between Bin 5 (Sequence B) and Bin 6 (Sequence A) to a similar level to as it was before the interference (Bin 4: 7.62 ms, 95% CI = [6.37, 8.87]; Bin 5: −1.49 ms, 95% CI = [−2.74, −0.25]; Bin 6: 7.81 ms, 95% CI = [6.56, 9.06]; Bin 4–5 contrast *p*_*cor*_ < 0.001, Bin 4–6 contrast *p*_*cor*_ = 0.995, Bin 5–6 contrast *p*_*cor*_ < 0.001). This indicates that participants were sensitive to the change in regularities, as suggested by the decrease in statistical learning performance in Bin 5. However, when exposed to the already-learned sequence again in Bin 6, the difference in RT between high- and low-probability triplets increased again, suggesting that the interfering information did not disrupt performance on the already-learned sequence (Fig. 5).

Neither the main effect of AQ (*F*(1, 822) < 0.001; *p* = 0.987), nor the interaction between Bin and AQ was significant (*F*(2, 822) = 0.107; *p* = 0.898). This suggests that AQ did not modulate the resistance to interfering statistical information in terms of reaction time (Fig. 5). The full list of fixed and random effect parameters can be found in Supplementary Table S3.

#### Analysis based on accuracy

**Fig. 6:**
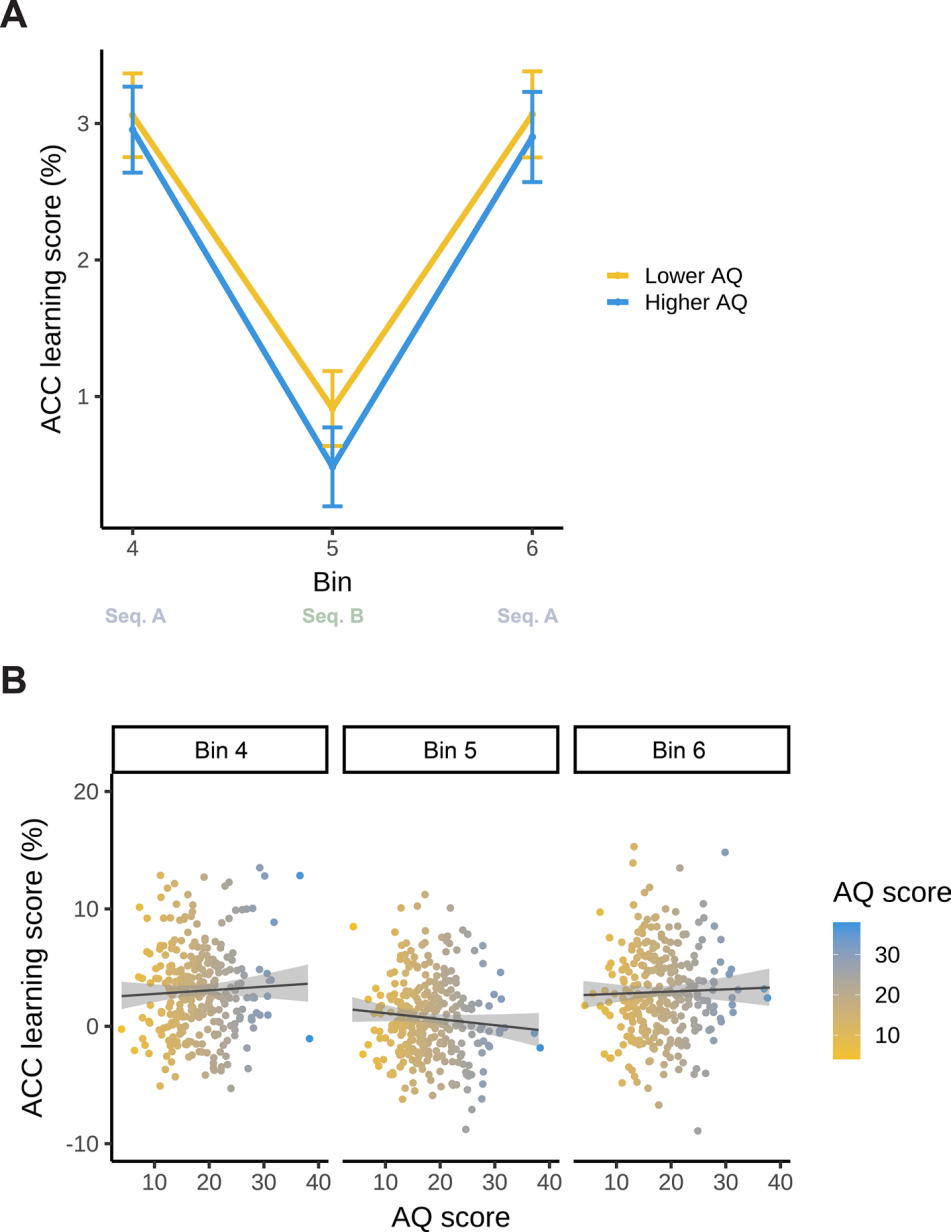
Model updating (accuracy) as a function of AQ. The y-axis represents performance in terms of binwise accuracy learning scores (the difference in binwise mean accuracy between high- and low-probability triplets). (**A)** Statistical learning trajectories are shown before (Bin 4), during (Bin 5), and after (Bin 6) exposure to interfering statistical information. Please note that AQ scores were entered in the analyses as continuous variables, but here, for interpretability, we also visualized AQ by median-splitting the sample into two groups. The yellow line represents lower AQ (scores 4–17), and the blue line represents higher AQ individuals (scores 18–38). The x-axis represents bins of the ASRT task (the progression of time). Error bars show 1 SEM. (**B)** Correlations between individual AQ sum scores and binwise accuracy learning scores are shown before (Bin 4), during (Bin 5), and after (Bin 6) exposure to interfering statistical information. The x-axis and the color bar represent AQ sum scores. The lines show the linear trend, and the shades reflect 1 SEM.

Model_U−ACC_ resulted in a statistically significant main effect of Bin (*F*(2, 588) = 38.671; *p* < 0.001). This indicates that statistical learning changed gradually during the task according to sequence identity (A or B): the difference in accuracy decreased between high- and low-probability triplets between Bin 4 (Sequence A) and Bin 5 (Sequence B), then increased again between Bin 5 (Sequence B) and Bin 6 (Sequence A) (Bin 4: 3.01%, 95% CI = [2.58, 3.43]; Bin 5: 0.69%, 95% CI = [0.28, 1.12]; Bin 6: 2.98%, 95% CI = [2.56, 3.41]; Bin 4–5 contrast *p*_*cor*_ < 0.001, Bin 4–6 contrast *p*_*cor*_ = 0.999, Bin 5–6 contrast *p*_*cor*_ < 0.001). These results are similar to those in the RT scores: the decrease in the difference in accuracy between high- and low-probability triplets in Bin 5 indicates participants’ sensitivity to the change in regularities. However, when exposed to the already-learned sequence again in Bin 6, the difference in accuracy between high-and low-probability triplets increased again. This suggests that the interfering information did not disrupt performance on the already-learned sequence (Fig. 6).

Neither the main effect of AQ (*F*(1, 294) = 0.022; *p* = 0.883), nor the interaction between Bin and AQ was significant (*F*(2, 588) = 1.548; *p* = 0.214). This suggests that AQ did not modulate the resistance to interfering statistical information in terms of accuracy (Fig. 6). The full list of fixed and random effect parameters can be found in Supplementary Table S4.

### Are autistic traits associated with a change in speed/accuracy trade-off?

Finally, to investigate the potential change in the trade-off between speed and accuracy throughout the task, we first aimed to ascertain the presence of SAT in the task and to examine its evolution. Thus, in Model_SAT_, we used RT as the outcome variable, adding Accuracy, Bin (1–6), and their interaction as fixed effects in the model. We specified random intercepts and random slopes for Bin for each participant. The main effect of Accuracy was significant (*F*(1, 1557) = 148.18; *p* < 0.001), indicating that participants were relatively slower when they were relatively more accurate (Estimate at 87.1% accuracy = 367 ms, 95% CI = [362, 371], estimate at 95.4% accuracy = 381 ms, 95% CI = [376, 385], high-low accuracy contrast *p*_*cor*_ < 0.001). The main effect of Bin was also significant (*F*(5, 1477) = 2.90; *p* = 0.013) indicating that participants became faster in subsequent Bins until they were exposed to interfering statistical information (but note that SAT itself is independent of statistical regularities), then became slower again (Bin 1: 392 ms, 95% CI = [387, 396]; Bin 2: 390 ms, 95% CI = [385, 395]; Bin 3: 380 ms, 95% CI = [375, 384]; Bin 4: 350 ms, 95% CI = [345, 355]; Bin 5: 366 ms, 95% CI = [362, 371]; Bin 6: 365 ms, 95% CI = [360, 370]; all pairwise contrasts between bins *p*_*cor*_ < 0.001, except for Bin 1–2 *p*_*cor*_ = 0.947 and Bin 5–6 *p*_*cor*_ = 0.992). Moreover, there was a trend-level interaction between Accuracy and Bin (*F*(5, 1477) = 2.11; *p* = 0.062). We do not report post-hoc tests for this model, but a descriptive figure of binwise correlations between speed and accuracy can be found in the Supplementary Materials (Figure S1). The full list of fixed and random effect parameters can be found in Supplementary Table S5.

**Fig. 7:**
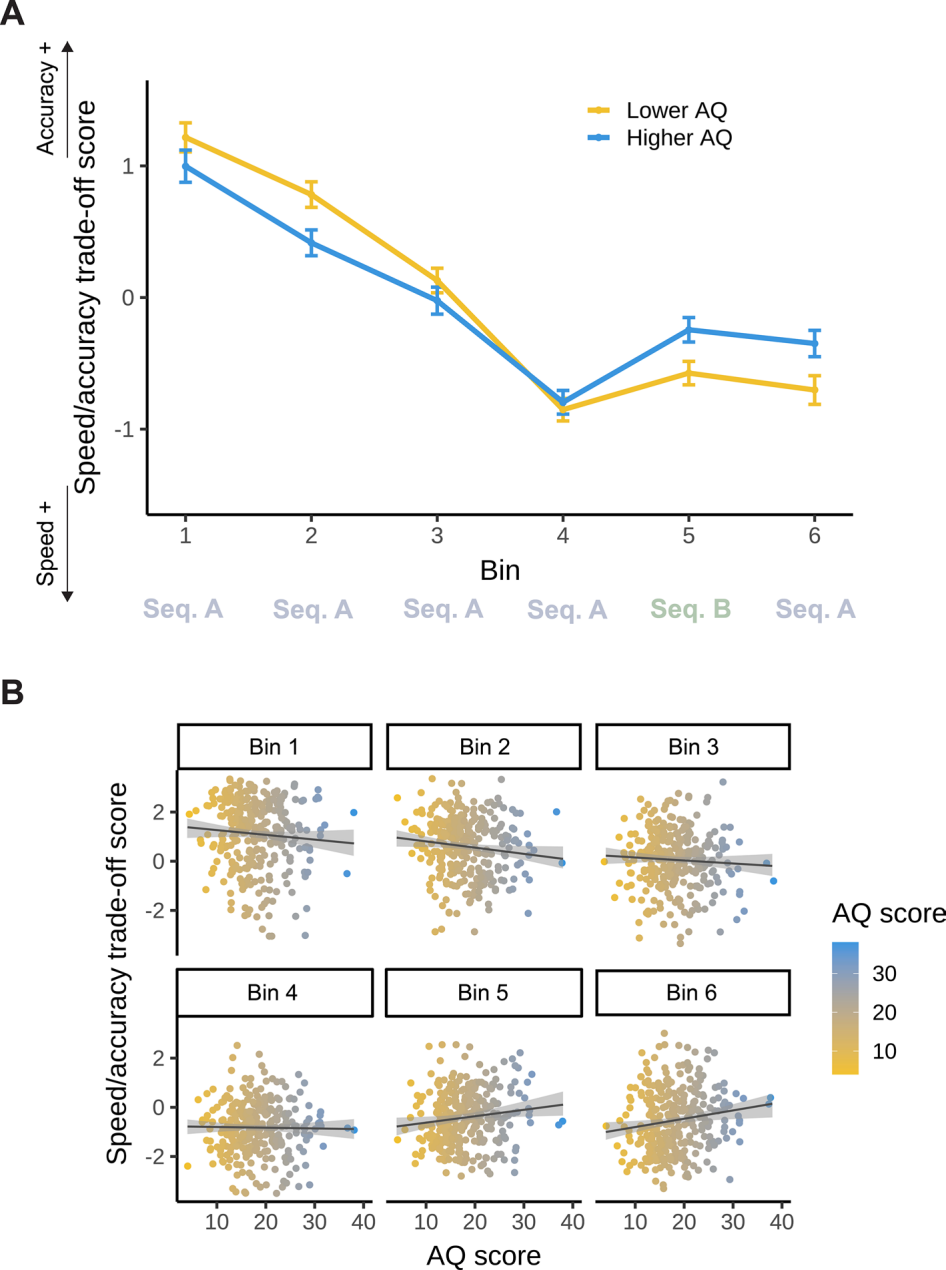
Speed/accuracy trade-off as a function of AQ in the ASRT task. The y-axis represents the binwise mean SAT score, which was calculated for each participant, indicating their relative response styles for each bin. Values close to zero indicate a relatively bias-free response style, more positive values indicate a more accurate response style at the expense of speed, while negative values indicate a faster response style at the expense of accuracy. **(A)** The trajectory of SAT is shown throughout the task (Bin 1–6). The x-axis represents bins of the ASRT task (the progression of time). Please note that AQ scores were entered in the analyses as continuous variables, but here, for interpretability, we also visualized AQ by median-splitting the sample into two groups. The yellow line represents lower AQ (scores 4–17), and the blue line represents higher AQ individuals (scores 18–38). Error bars show 1 SEM. (**B)** Correlations between individual AQ sum scores and binwise SAT scores are shown throughout the task (Bin 1–6). The x-axis and the color bar represent AQ sum scores. The lines show the linear trend, and the shades reflect 1 SEM.

After obtaining evidence that SAT was present in our task, we next sought to investigate whether and how autistic traits related to this measure. We fit a linear mixed model with trade-off score (see above) as the outcome, including Bin (1–6), AQ, and their interaction as fixed effects in the model. We specified random intercepts and random slopes for Bin for each participant. Model_SAT−AQ_ resulted in a statistically significant main effect of Bin (*F*(5, 1764) = 109.22; *p* < 0.001), indicating that participants became gradually more biased toward speed until they were exposed to interfering statistical information (but note that SAT itself is independent of statistical regularities), then became less biased again (Bin 1: 1.11, 95% CI = [0.97, 1.25]; Bin 2: 0.60, 95% CI = [0.46, 0.74]; Bin 3: 0.05, 95% CI = [−0.08, 0.19]; Bin 4: −0.82, 95% CI = [−0.96, −0.69]; Bin 5: −0.41, 95% CI = [−0.55, −0.27]; Bin 6: −0.53, 95% CI = [−0.67, −0.39]; all pairwise contrasts between bins *p*_*cor*_ < 0.001, except for Bin 4–6 *p*_*cor*_ = 0.043, Bin 5–6 *p*_*cor*_ = 0.985) (Fig. 7). The main effect of AQ was not significant (*F*(1, 1764) < 0.001; *p* > 0.999). However, the interaction between Bin and AQ was significant (*F*(5, 1764) = 3.85; *p* = 0.002), indicating that AQ related to the evolution of SAT throughout the task (Fig. 7). In other words, the trajectory of overall SAT differed as a function of AQ. Based on the model estimates, individuals with a higher AQ score became less biased toward speed after a being exposed to interfering statistical information (see estimates and contrasts in Supplementary Table S6 and S7, respectively). The full list of fixed and random effect parameters can be found in Supplementary Table S8.

## Discussion

The predictive processing framework of ASD suggests alterations in how autistic individuals rely on internal models constructed from prior experience to predict future events^3,4^. These alterations are assumed to be at the root of autistic symptoms such as difficulties in adaptation. Here, we tested the slow updating hypothesis, an approach within the predictive processing framework of ASD that suggests a slower updating of internal models in autistic individuals^14^. However, we did not measure ASD participants but applied the spectrum approach of ASD, which promotes the detection of inter-individual variability through the use of large samples. This approach may also enable the detection of alterations at the root of the condition, potentially even without the symptoms unfolding into a disorder. It may help better understand processes that are shared among all autistic individuals, a highly heterogeneous condition. In that vein, we applied the spectrum approach of ASD, recruiting a large sample of 296 adults and assessing their degree of autistic traits. We measured predictive processing by a widely used implicit statistical learning task^45^. Furthermore, to control for the potential confounding effect of a difference in visuomotor performance along autistic traits, we assessed speed/accuracy trade-off and its relationship with autistic traits in the same experimental design. We found comparable performance in both statistical learning and model updating rate along autistic traits, suggesting no relationship between such traits and model updating. In contrast, our results show a difference in the evolution of speed/accuracy trade-off in the task along the degree of autistic traits.

Our results showing comparable performance in the acquisition of statistical information along autistic traits is in line with several studies showing intact statistical learning in ASD^46,57,79–84^. Our study adds to this body of evidence by focusing on individual differences involving a large sample of 296 neurotypical individuals under the framework of the spectrum approach of ASD^26–28^. By quantifying autistic traits, we were able to treat them as a continuous variable and, thus, go beyond the dichotomic comparison of groups by performing fine-grained, multivariate analyses with the assumption of large variability. Specifically, we fit linear mixed models on our data to investigate the relationship of autistic traits with the cognitive processes in question. Our models were fit with a random intercept for each participant, accounting for the inter-individual variability in our data. The spectrum approach goes beyond traditional case-control studies, which is especially significant in the case of such a vastly heterogeneous condition^16–18^, where the presence of a diagnosis in itself fails to capture the diversity of traits and symptoms of each individual (although our sample did not capture the entire spectrum). In summary, our results within the spectrum framework in the general population showing no difference corroborate previous findings on statistical learning in diagnosed groups. Nevertheless, it leaves an open question about where the atypicalities of predictive processing in ASD can be grasped.

The comparable performance in model updating rate along autistic traits was not in line with the slow updating approach. There are multiple possible explanations for this result. Firstly, it is possible that the slow updating approach only holds for individuals with a high degree of autistic traits, usually also with a diagnosis of ASD. And since we recruited a non-diagnosed sample, the distribution of AQ scores in this sample represents the distribution typically seen in the neurotypical population^23^, with only a few individuals on the high extreme of the spectrum, as expected. Specifically, only 3 of the 296 individuals (1%) scored above the threshold of 32 points, a proxy for diagnosis proposed by Baron-Cohen et al.^52^. If slow updating were only observable in the diagnosed population or at a higher degree of autistic traits, it is possible that the lack of extreme high scores on the AQ scale obscures this effect in our study. This would, however, suggest a qualitative rather than quantitative difference between individuals with a diagnosis or above the AQ threshold and individuals on the rest of the spectrum, which would not align with the spectrum framework. Alternatively, there might be a non-linear relationship between statistical learning abilities and AQ scores, where the slope remains nearly flat across lower AQ scores (i.e., below 32, as observed in our population), but may increase more steeply as AQ scores approach the higher end of the spectrum (up to 50). Future studies should include a broader range of AQ scores to more thoroughly investigate this relationship.

Another consideration is that our approach relied exclusively on surface-level behavioral indices such as reaction time, accuracy, and speed/accuracy trade-off. While these measures can reflect certain aspects of learning and decision-making, they may not sufficiently capture the latent computational processes that underlie predictive coding – such as dynamic prior weighting, learning rates, or volatility estimation. Thus, the lack of observed differences in model updating may reflect limitations in measurement sensitivity rather than genuine equivalence in processing. In particular, predictive coding theories often conceptualize internal neural noise or uncertainty in terms of precision-weighted prediction errors (e.g^15,85^.,). From this perspective, behavioral variability – such as trial-to-trial fluctuations in response time – has been proposed as a potential proxy for internal noise or imprecise inference (e.g^13^.,). While variability-based metrics were not analyzed in the current study, acknowledging this dimension clarifies the scope of our findings and highlights an important direction for future research. Additionally, neural noise may offer an alternative explanation for the current findings. Rather than reflecting truly similar learning speeds across levels of autistic traits, the absence of differences could instead reflect effects that are masked by greater internal noise – making learning harder to detect through mean-level measures. Likewise, the SAT pattern we observed could be interpreted as a compensatory shift in strategy, with individuals adopting more accuracy-focused responses to counteract higher internal uncertainty. Future work would benefit from applying computational modeling approaches, such as hierarchical Bayesian learning models, which can extract estimates of internal states like precision-weighted prediction errors, priors, and learning rates (e.g^86,87^.,). Such models may also be able to disentangle meaningful signal from noise and offer a more fine-grained test of precision-weighted inference. These approaches would allow for a more process-level test of predictive processing theories and clarify the mechanisms by which autistic traits may influence learning under uncertainty.

Visuomotor skills are required while performing ASRT task, therefore, to control for a potential confounding effect, we assessed whether autistic traits were related to differences in visuomotor performance on the task, separately from statistical learning. As a measure of visuomotor performance, we calculated a trade-off score between speed and accuracy (SAT), which provides a more complex insight into visuomotor decision-making than either speed or accuracy on its own. In ASD, there is evidence for neural alterations related to visuomotor processing^36,37^, with ambiguous results on the behavioral level^34,35,46,51^. Based on results on ASD and visuomotor performance on the ASRT by Pesthy et al.^46^ and Nagy et al.^51^, we expected autistic traits to have no association with speed/accuracy trade-off. However, we found that the trajectory of overall SAT differed along autistic traits. Specifically, individuals with a higher degree of autistic traits became less biased toward speed (i.e., more balanced in their speed and accuracy) compared to individuals with a lower degree of autistic traits, after the sequence in the task changed. Individuals with a lower degree of autistic traits remained significantly speedier at the expense of accuracy, compared to their own baseline.

A speculative explanation for the difference in the trajectory of SAT along autistic traits could involve the potential shift in the balance of goal-directed and habitual systems (for more on the relationship of these systems, see^88–90^) in association with autistic traits. In particular, a bias toward accuracy could mirror a more goal-directed, attentive response style, while a bias toward speed could reflect a more automatic, habitual behavior^91,92^. So, it can be speculated that when the learned pattern of the environment changes, the behavior of individuals with more autistic traits becomes less automatic. It is possible that more salience is ‘attributed’ in a non-conscious way to the fact that something has changed in the environment, and what had become a habit is not sufficient anymore. Interestingly, in a perceptual learning task^93^, only some autistic adults (but no neurotypicals) explicitly noticed subtle contextual changes when statistical regularities shifted. Yet, in their study, autistic individuals were slower at updating their priors despite detecting changes in regularities. Even though the SAT interpretation of the current study is speculative, it sets the stage for future research on the interplay between executive functions and predictive, habitual processes in ASD, and how these relate to autistic symptoms, for example, rigidity. This line of investigation could be especially relevant given that altered executive functioning is also a candidate framework to explain autistic symptoms^94,95^. Furthermore, SAT provides an opportunity to dissociate visuomotor performance from predictive model-building, promoting a more process-pure examination. Based on all this, it could be a promising aspect to further investigate in statistical learning and could have substantial methodological significance in the future.

Another possibility is that individuals with higher autistic traits engaged in more cautious, accuracy-prioritizing response strategies throughout the task – strategies that may reflect more explicit control over performance, even in an implicit learning context. Although our task did not provide performance feedback, participants could likely detect when they made an error, which may have led some to consciously adjust their speed in order to reduce errors. This is in line with the notion of ‘response caution’, whereby individuals sensitive to errors deliberately slow down to increase accuracy^96^. Such a strategy may be more prevalent among individuals high in autistic traits: studies found alteration in response caution among individuals with autistic spectrum disorders, although varying in the direction of the effect^97–100^. Thus, the SAT pattern we observed may reflect not only underlying cognitive system dynamics (i.e., goal-directed vs. habitual) but also more explicit, performance-monitoring strategies. Future work could explore the extent to which such strategies are deployed, potentially using verbal reports, confidence ratings, or trial-by-trial feedback manipulations.

Another potential explanation for the altered evolution of SAT as a function of autistic traits can be related to the findings of an overestimation of volatility in ASD^13^. According to these findings, when autistic individuals face changes in the environment, they overestimate the volatility of that environment at the expense of learning about probabilistically atypical events that do not fit the learned regularity (i.e., exceptions to the rule). Besides computational modeling and pupillometry data, this conclusion was based on behavioral results indicating a difference between ASD and neurotypical participants in accuracy and reaction time as a function of volatility^13^. Specifically, when beliefs about phasic volatility increased, neurotypical participants became faster^13^ and more accurate. Autistic individuals, however, became slower but remained similarly accurate – i.e., their speed/accuracy trade-off became more balanced. This corresponds to what we see in the results of this study in association with autistic traits, as volatility can be captured as a change in the learned pattern of the environment (i.e., the change from Sequence A to Sequence B). The question remains open, however, as to why this behavioral measure, which might be an indicator of the overestimation of volatility in relation to high autistic traits, is only manifest in visuomotor performance and not in statistical learning.

From a neural perspective, SAT shifts in cognitive tasks have been associated with mind-wandering^66,101^, which is linked to the activity of the brain’s default mode network^102–104^. In a broader context, the default mode network seems to be related to functions such as internally originated cognition, constructive mental simulations, and mentalizing^103^, and is widely acknowledged to be atypical in ASD (for a review, see^105^). These differences in the default mode network in association with ASD could potentially induce atypicalities in internally oriented mental processes, such as mind-wandering. Thus, altered mind-wandering processes could underlie the difference in SAT trajectory along the autistic spectrum. Furthermore, sleep disturbances are prominent in ASD^106–110^, which could also cause alterations in mind-wandering^111,112^. Therefore, future research could employ techniques like electroencephalography (EEG) or magnetoencephalography (MEG) to monitor the activity and connectivity of the default mode network in association with SAT, mind-wandering, and autistic traits.

While the above interpretations focus on trait-linked mechanisms and strategic adaptations, it is also important to consider that SAT changes over the course of the task may reflect more general, non-specific influences. In long-duration, repetitive tasks like the ASRT, factors such as task fatigue, reduced motivation, or attentional disengagement could contribute to observed changes in SAT, particularly toward the later bins^113,114^. These state-related factors might mimic or obscure trait-related differences. While here, we did not directly measure subjective engagement, incorporating self-report effort ratings or periodic probes of task motivation and fatigue in future studies could help dissociate such general influences from more specific cognitive control processes.

A limitation of this work could be the use of a single measurement to assess the degree of autistic traits. Even though the AQ is a widely used and validated instrument used both in research and practice^53,115,116^, it could be worth employing a wider range of questionnaires to cover even more aspects of the heterogeneous condition of ASD. Examples for such additional measures are the Social Responsiveness Scale-2 (SRS-2)^117^ or the Adult Repetitive Behaviours Questionnaire-2 (RBQ-2A)^118^ to assess ASD symptoms, the Ritvo Autism Asperger Diagnostic Scale-Revised (RAADS-R)^119^ the Camouflaging Autistic Traits Questionnaire (CAT-Q)^120^ to assess autistic traits, or the Mentalization Questionnaire (MZQ)^121^ to assess the ability to understand and interpret one’s own and others’ mental states. An important strength of this work is the use of the spectrum approach. It overcomes the constraints of patient recruitment and allows for the use of a large sample where inter-individual differences can be detected using multivariate analyses. However, a drawback is that it only assesses a narrow range of autistic traits in the general population. Addressing variability is particularly significant for ASD, a highly heterogeneous condition^16–18^, where the presence of a diagnosis in itself fails to capture the diversity of traits and symptoms of each individual. Studies in the spectrum framework may also be less affected by confounding factors like comorbidities and medication.

Our study highlights a fundamental question in neuropsychiatry regarding the nature of individual differences in ASD: is there a quantitative or qualitative distinction between diagnosed individuals with ASD and those with high autistic traits who remain undiagnosed? The spectrum approach, which emphasizes the continuous nature of autistic traits, suggests that the difference is primarily quantitative^26–28^. According to this view, individuals with elevated scores on measures like the AQ share similarities with those diagnosed with ASD, indicating that autism lies on a continuum rather than being a distinct category. However, if significant differences are found between undiagnosed individuals with high AQ scores and those diagnosed with ASD who display comparable AQ scores, this would suggest a qualitative difference, raising doubts about the validity of the purely quantitative spectrum model or of the AQ as a tool to measure autistic traits on the spectrum. Such differences could point to unique clinical or neurobiological factors that distinguish diagnosed individuals from those with subclinical traits, calling for a more nuanced understanding of ASD that accounts for both dimensions. Future research that combines diagnosed ASD and neurotypical samples with a broad range of AQ scores could provide crucial insights into whether these differences are best conceptualized as variations along a spectrum or as fundamentally distinct phenomena. Understanding this distinction will have important implications for diagnosis, treatment, and how we define neurodiversity.

It is plausible that a longer exposure to interfering information (Sequence B) could provide more opportunity for variations in the updating rate to emerge among participants with different AQ scores. In our task, to minimize participant burden, the interfering sequence was relatively short, lasting only around 5–6 min. This brevity raises the possibility that we may not have adequately captured the full scope of the updating process, as the limited number of data points might have been insufficient to reveal meaningful differences. Although increasing the length of the task might introduce a slight burden on participants, it would be a valuable modification for future studies. Extending the task could allow for a more comprehensive assessment of model updating, providing a clearer picture of how individuals with varying levels of autistic traits respond to interference. Trial-by-trial computational modeling of the interference phase could also reveal more subtle differences in the updating of statistical regularities across autistic traits. To enhance the robustness of these findings, future work could consider administering the ASRT task or other statistical learning tasks across multiple sessions^48,122^. This approach would not only mitigate participant fatigue^123^, but also create more opportunities to observe potential differences in updating processes underlying individual differences.

Anticipatory processes and error analysis are crucial for unlocking a deeper understanding of predictive processing in ASD. A new, innovative version of the sequence learning task uses eye tracking to measure anticipatory eye movements, that is, gaze shifts during response-to-stimulus intervals^124,125^. These anticipatory eye movements directly reflect priors, as they are made when no sensory stimulus is present on the screen. The analysis of errors made in these expectations can provide a sophisticated, in-depth insight into the creation and updating of the internal models of participants. The probabilistic nature of the ASRT task allows for the distinction of two types of errors: learning-dependent errors and not-learning-dependent errors. Learning-dependent errors reflect an expectation that aligns with the probability structure in the task (i.e., learning), but they occur when a low-probability stimulus appears by chance, hence the incorrect expectation. On the other hand, not-learning-dependent errors occur when the expectation corresponds to a low-probability stimulus, thus they do not reflect learning. The distinction of these errors and how they induce the adjusting of expectations (i.e., priors) offers a more refined inference about internal model updating, whereby potential nuances along the autistic spectrum could emerge. Future studies testing these hypotheses and their links to autistic traits with eye-tracking methodology seem highly warranted.

In conclusion, we found no alterations in statistical learning or model updating rate along autistic traits in the general population. However, we found that a higher degree of autistic traits was associated with different visuomotor decision-making dynamics compared to a lower degree of autistic traits after the pattern in the environment changed. These results offer inspiration for future studies from conceptual, methodological, and applied aspects. Conceptually, future studies should combine the spectrum approach with a neurodivergent sample in the investigation of predictive processing to pinpoint how autistic traits and diagnoses relate to cognitive functioning. Methodologically, the assessment of speed/accuracy trade-off in statistical learning offers a process-pure yet complex measurement of visuomotor decision-making that should be further exploited by future research. Regarding applied implications, if interpreted in the context of clinical ASD, intact statistical learning and model updating functions could provide a basis for strength-based interventions.

## Supplementary Information

Below is the link to the electronic supplementary material.

Supplementary Material 1

## Data Availability

All data and codes for data preprocessing, analysis, and visualization are available at: https://osf.io/5tsgy/.
