# Supplementary material for: Autistic traits relate to speed/accuracy trade-off but not statistical learning and updating
Source: Sci Rep. 2025 Aug 30;15:32001. doi: 10.1038/s41598-025-16138-7 (PMC12398567; doi:10.1038/s41598-025-16138-7)
Supplement: Supplementary file 1 — Supplementary Material 1 [file 41598_2025_16138_MOESM1_ESM.docx]

**Supplementary Materials** for the manuscript entitled

**‘Autistic Traits Relate to Speed/Accuracy Trade-off but not Statistical Learning and Updating’**

**Flóra Hann^1,2,3,4^, Orsolya Pesthy^4,5^, Bianka Brezóczki^1,2,4^, Teodóra Vékony^5,6^, Cintia Anna Nagy^2^, Laurie-Anne Sapey-Triomphe^5^, Eszter Tóth-Fáber^2,4^, Bence Csaba Farkas^7,8,9^, Kinga Farkas^10^, Dezső Németh^5,6,11^**

*^1^Doctoral School of Psychology, ELTE Eötvös Loránd University, Budapest, Hungary*

*^2^Institute of Psychology, ELTE Eötvös Loránd University, Budapest, Hungary*

*^3^Institute of Experimental Medicine, HUN-REN Research Centre for Natural Sciences, Budapest, Hungary*

*^4^Institute of Cognitive Neuroscience and Psychology, HUN-REN Research Centre for Natural Sciences, Budapest, Hungary*

*^5^Centre de Recherche en Neurosciences de Lyon CRNL U1028 UMR5292, INSERM, CNRS, Université Claude Bernard Lyon 1, Bron, France*

*^6^Department of Education and Psychology, Faculty of Social Sciences, University of Atlántico Medio, Las Palmas de Gran Canaria, Spain*

*^7^Institut du Psychotraumatisme de l’Enfant et de l’Adolescent, Conseil Départemental Yvelines et Hauts-de-Seine et Centre Hospitalier des Versailles, Versailles, France*

*^8^UVSQ, Inserm, Centre de Recherche en Epidémiologie et Santé des Populations, Université Paris-Saclay, Versailles, France*

*^9^LNC2, Département d’études Cognitives, École Normale Supérieure, INSERM, PSL Research University, Paris, France*

*^10^Department of Psychiatry and Psychotherapy, Semmelweis University, Budapest, Hungary*

*^11^BML-NAP Research Group, Institute of Psychology, Eötvös Loránd University and Institute of Cognitive Neuroscience and Psychology, HUN-REN Research Centre for Natural Sciences, Budapest, Hungary*

### Participants and Screening

**Supplementary Text S1.** Interaction effects from models including the two individuals who self-reported an ASD diagnosis and were excluded from the final sample.

Model_SL-RT_ Bin × AQ: *F*_2, 592_ = 0.10; *p* = .902

Model_SL-ACC_ Bin × AQ: *F*_2, 592_ = 0.38; *p* = .686

Model_U-RT_ Bin × AQ: *F*_2, 888_ = 0.21; *p* = .809

Model_U-ACC_ Bin × AQ: *F*_2, 592_ = 1.83; *p* = .162

Model_SAT-AQ_ Bin × AQ: *F*_5, 1776_ = 3.54; ***p* = .004**

### Model_SL-RT_

**Supplementary Table S1.** Results of the linear mixed model predicting RT learning scores in the learning phase.

| **Prediction of RT Learning Score** | | | | | |
| --- | --- | --- | --- | --- | --- |
| **Fixed Effects** | ***β estimates*** | ***95% CI*** | ***t*** | ***df*** | ***p*** |
| **(Intercept)** | 2.622 | 1.020 – 4.224 | 3.212 | 876.178 | **.001** |
| Bin [2] | 1.459 | -0.742 – 3.660 | 1.302 | 588.000 | .193 |
| **Bin [3]** | 5.034 | 2.832 – 7.235 | 4.491 | 588.000 | **< .001** |
| AQ | 0.003 | -0.281 – 0.287 | 0.020 | 876.178 | .984 |
| Bin [2] × AQ | 0.044 | -0.345 – 0.434 | 0.223 | 588.000 | .823 |
| Bin [3] × AQ | 0.022 | -0.367 – 0.412 | 0.112 | 588.000 | .911 |
| **Random Effects** | | | | | |
| σ^2^ | 185.902 | | | | |
| τ_00_ _id_ | 11.371 | | | | |
| ICC | 0.058 | | | | |
| N _id_ | 296 | | | | |
| Observations | 888 | | | | |
| Marginal R^2^ / Conditional R^2^ | 0.022 / 0.079 | | | | |

The table shows regression coefficients of fixed effects and summary information of random effects. The marginal R^2^ considers only the variance of fixed effects, while the conditional R^2^ takes both fixed and random effects into account. Degrees of freedom are based on Satterthwaite’s approximation. Statistically significant terms are highlighted in bold. Terms in brackets indicate the level of factor that is contrasted against the reference level, which is Bin 1 for the Bin factor.

### Model_SL-ACC_

**Supplementary Table S2.** Results of the linear mixed model predicting accuracy learning scores (%) in the learning phase.

| **Prediction of Accuracy Learning Score** | | | | | |
| --- | --- | --- | --- | --- | --- |
| **Fixed Effects** | ***β estimates*** | ***95% CI*** | ***t*** | ***df*** | ***p*** |
| **(Intercept)** | 1.376 | 0.947 – 1.805 | 6.298 | 874.676 | **< .001** |
| **Bin [2]** | 0.773 | 0.187 – 1.360 | 2.588 | 588.000 | **.010** |
| **Bin [3]** | 1.795 | 1.208 – 2.382 | 6.004 | 588.000 | **< .001** |
| AQ | -0.027 | -0.103 – 0.049 | -0.692 | 874.676 | .489 |
| Bin [2] × AQ | 0.059 | -0.045 – 0.163 | 1.109 | 588.000 | .268 |
| Bin [3] × AQ | 0.031 | -0.073 – 0.135 | 0.590 | 588.000 | .556 |
| **Random Effects** | | | | | |
| σ^2^ | 13.220 | | | | |
| τ_00_ _id_ | 0.915 | | | | |
| ICC | 0.065 | | | | |
| N _id_ | 296 | | | | |
| Observations | 888 | | | | |
| Marginal R^2^ / Conditional R^2^ | 0.038 / 0.100 | | | | |

The table shows regression coefficients of fixed effects and summary information of random effects. The marginal R^2^ considers only the variance of fixed effects, while the conditional R^2^ takes both fixed and random effects into account. Degrees of freedom are based on Satterthwaite’s approximation. Statistically significant terms are highlighted in bold. Terms in brackets indicate the level of factor that is contrasted against the reference level, which is Bin 1 for the Bin factor.

### Model_U-RT_

**Supplementary Table S3.** Results of the linear mixed model predicting RT learning scores in the interference phase.

| **Prediction of RT Learning Score** | | | | | |
| --- | --- | --- | --- | --- | --- |
| **Fixed Effects** | ***β estimates*** | ***95% CI*** | ***t*** | ***df*** | ***p*** |
| **(Intercept)** | 7.620 | 6.370 – 8.870 | 11.963 | 882.000 | **< .001** |
| **Bin [5]** | -9.115 | -10.882 – -7.347 | -10.118 | 882.000 | **< .001** |
| Bin [6] | 0.191 | -1.577 – 1.959 | 0.212 | 882.000 | .832 |
| AQ | 0.042 | -0.180 – 0.263 | 0.369 | 882.000 | .712 |
| Bin [5] × AQ | -0.064 | -0.377 – 0.249 | -0.404 | 882.000 | .686 |
| Bin [6] × AQ | -0.064 | -0.377 – 0.249 | -0.399 | 882.000 | .690 |
| **Random Effects** | | | | | |
| σ^2^ | 120.094 | | | | |
| τ_00_ _id_ | 0.000 | | | | |
| N _id_ | 296 | | | | |
| Observations | 888 | | | | |
| Marginal R^2^ / Conditional R^2^ | 0.136 / NA | | | | |

The table shows regression coefficients of fixed effects and summary information of random effects. The marginal R^2^ considers only the variance of fixed effects, while the conditional R^2^ takes both fixed and random effects into account. Degrees of freedom are based on Satterthwaite’s approximation. Statistically significant terms are highlighted in bold. Terms in brackets indicate the level of factor that is contrasted against the reference level, which is Bin 4 for the Bin factor.

### Model_U-ACC_

**Supplementary Table S4.** Results of the linear mixed model predicting accuracy learning scores (%) in the interference phase.

| **Prediction of Accuracy Learning Score** | | | | | |
| --- | --- | --- | --- | --- | --- |
| **Fixed Effects** | ***β estimates*** | ***95% CI*** | ***t*** | ***df*** | ***p*** |
| **(Intercept)** | 3.008 | 2.584 – 3.431 | 13.934 | 880.979 | **< .001** |
| **Bin [5]** | -2.308 | -2.901 – -1.716 | -7.655 | 588.000 | **< .001** |
| Bin [6] | -0.023 | -0.616– 0.569 | -0.078 | 588.000 | .938 |
| AQ | 0.031 | -0.044 – 0.106 | 0.806 | 880.979 | .421 |
| Bin [5] × AQ | -0.082 | -0.187 – 0.023 | -1.530 | 588.000 | .126 |
| Bin [6] × AQ | -0.001 | -0.106 – 0.104 | -0.013 | 588.000 | .989 |
| **Random Effects** | | | | | |
| σ^2^ | 13.459 | | | | |
| τ_00_ _id_ | 0.332 | | | | |
| ICC | 0.024 | | | | |
| N _id_ | 296 | | | | |
| Observations | 888 | | | | |
| Marginal R^2^ / Conditional R^2^ | 0.081 / 0.103 | | | | |

The table shows regression coefficients of fixed effects and summary information of random effects. The marginal R^2^ considers only the variance of fixed effects, while the conditional R^2^ takes both fixed and random effects into account. Degrees of freedom are based on Satterthwaite’s approximation. Statistically significant terms are highlighted in bold. Terms in brackets indicate the level of factor that is contrasted against the reference level, which is Bin 4 for the Bin factor.

### Model_SAT_

###
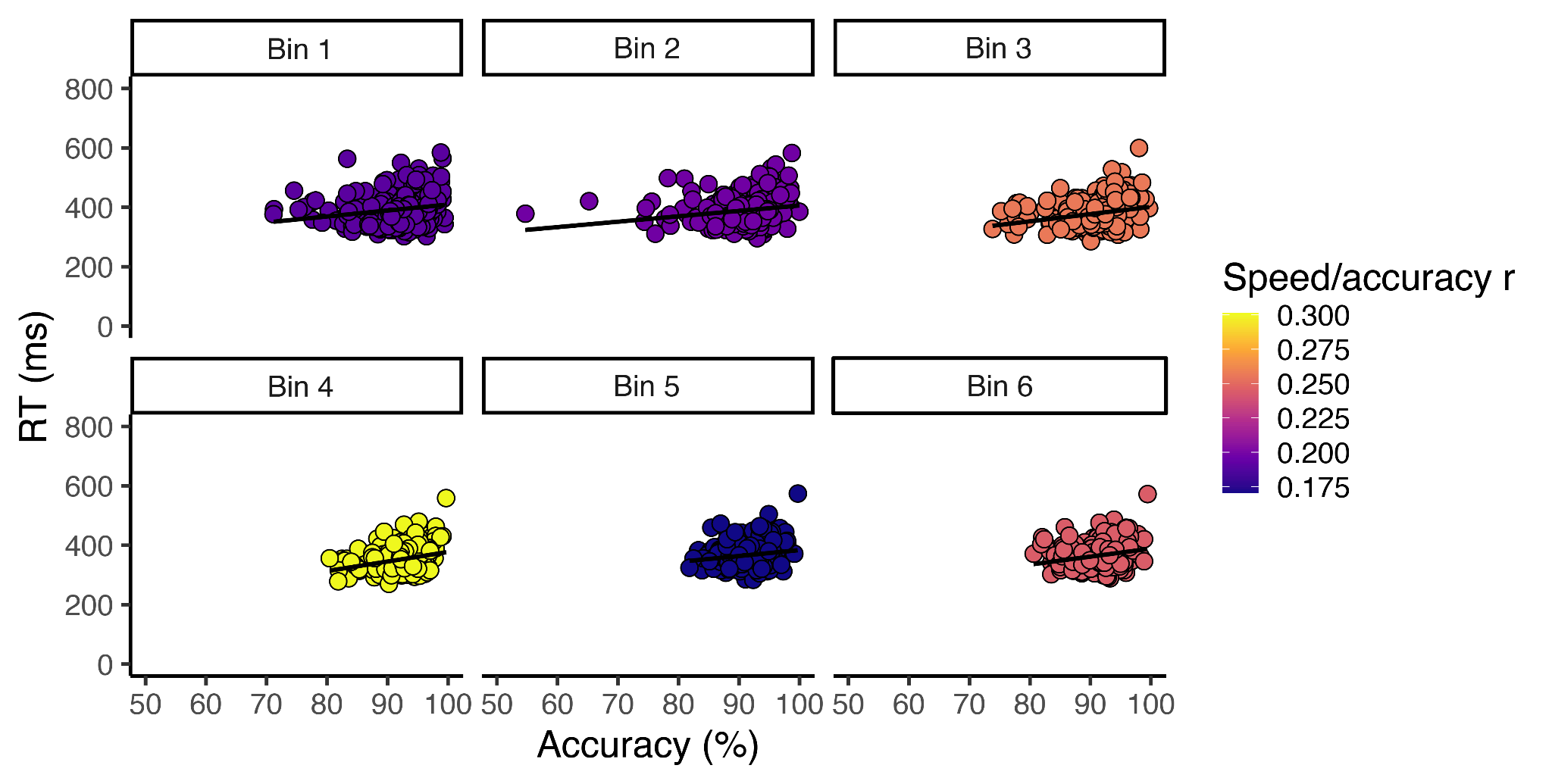


**Supplementary Figure S1.** Binwise correlations between speed and accuracy.

**Supplementary Table S5.** Results of the linear mixed model predicting binwise median RT throughout the task.

| **Prediction of Binwise Median RT** | | | | | |
| --- | --- | --- | --- | --- | --- |
| **Fixed Effects** | ***β estimates*** | ***95% CI*** | ***t*** | ***df*** | ***p*** |
| **(Intercept)** | 202.594 | 164.914 – 240.273 | 10.547 | 1535.876 | **<0.001** |
| **Bin [2]** | 67.294 | 19.117 – 115.472 | 2.740 | 1475.833 | **0.006** |
| Bin [3] | 32.766 | -18.402 – 83.935 | 1.256 | 1477.257 | 0.209 |
| Bin [4] | -31.749 | -90.887 – 27.388 | -1.053 | 1479.523 | 0.292 |
| Bin [5] | 37.410 | -24.686 – 99.506 | 1.182 | 1480.329 | 0.237 |
| Bin [6] | 14.378 | -44.820 – 73.575 | 0.476 | 1479.208 | 0.634 |
| **Binwise Mean Accuracy** | 2.072 | 1.665 – 2.480 | 9.973 | 1496.976 | **<0.001** |
| **Bin [2] × Binwise Mean Accuracy** | -0.756 | -1.284 – -0.229 | -2.813 | 1475.853 | **0.005** |
| Bin [3] × Binwise Mean Accuracy | -0.491 | -1.052 – 0.070 | -1.716 | 1477.332 | 0.086 |
| Bin [4] × Binwise Mean Accuracy | -0.106 | -0.749 – 0.537 | -0.324 | 1479.507 | 0.746 |
| **Bin [5] × Binwise Mean Accuracy** | -0.688 | -1.367 – -0.009 | -1.988 | 1480.397 | **0.047** |
| Bin [6] × Binwise Mean Accuracy | -0.450 | -1.099 – 0.198 | -1.364 | 1479.290 | 0.173 |
| **Random Effects** | | | | | |
| σ^2^ | 230.116 | | | | |
| τ_00_ _id_ | 1483.701 | | | | |
| ICC | 0.866 | | | | |
| N _id_ | 296 | | | | |
| Observations | 1776 | | | | |
| Marginal R^2^ / Conditional R^2^ | 0.130 / 0.883 | | | | |

The table shows regression coefficients of fixed effects and summary information of random effects. The marginal R^2^ considers only the variance of fixed effects, while the conditional R^2^ takes both fixed and random effects into account. Degrees of freedom are based on Satterthwaite’s approximation. Statistically significant terms are highlighted in bold. Terms in brackets indicate the level of factor that is contrasted against the reference level, which is Bin 1 for the Bin factor.

### Model_SAT-AQ_

**Supplementary Table S6.** Estimates for the mean trade-off score in each bin at 1 SD below (‘low’) and 1 SD above (‘high’) the mean AQ score.

| **AQ** | **Bin** | **Estimated Mean Trade-off Score** | ***95% CI*** |
| --- | --- | --- | --- |
| 12.45 (‘low’) | 1 | 1.217 | 1.022 – 1.413 |
|  | 2 | 0.744 | 0.548 – 0.939 |
|  | 3 | 0.123 | -0.073 – 0.318 |
|  | 4 | -0.807 | -1.003 – -0.612 |
|  | 5 | -0.557 | -0.754 – -0.362 |
|  | 6 | -0.718 | -0.913 – -0.522 |
| 23.75 (‘high’) | 1 | 0.999 | 0.803 – 1.195 |
|  | 2 | 0.457 | 0.261 – 0.653 |
|  | 3 | -0.016 | -0.211 – 0.180 |
|  | 4 | -0.841 | -1.037 – -0.645 |
|  | 5 | -0.263 | -0.459 – -0.068 |
|  | 6 | -0.336 | -0.532 – -0.140 |

Please note that AQ scores were entered in the analyses as continuous variables, but here, for interpretability, we compute estimates for scores 1 SD below and above the mean.

**Supplementary Table S7.** Contrasts between estimates for the mean trade-off score in each bin at 1 SD below (‘low’) and 1 SD above (‘high’) the mean AQ score.

| **Contrast** | ***p*** |
| --- | --- |
| Bin 1, low AQ – Bin 2, low AQ | .052 |
| Bin 1, low AQ – Bin 3, low AQ | **< .001** |
| Bin 1, low AQ – Bin 4, low AQ | **< .001** |
| Bin 1, low AQ – Bin 5, low AQ | **< .001** |
| Bin 1, low AQ – Bin 6, low AQ | **< .001** |
| Bin 1, low AQ – Bin 1, high AQ | .999 |
| Bin 1, low AQ – Bin 2, high AQ | **< .001** |
| Bin 1, low AQ – Bin 3, high AQ | **< .001** |
| Bin 1, low AQ – Bin 4, high AQ | **< .001** |
| Bin 1, low AQ – Bin 5, high AQ | **< .001** |
| Bin 1, low AQ – Bin 6, high AQ | **< .001** |
| Bin 2, low AQ – Bin 3, low AQ | **< .001** |
| Bin 2, low AQ – Bin 4, low AQ | **< .001** |
| Bin 2, low AQ – Bin 5, low AQ | **< .001** |
| Bin 2, low AQ – Bin 6, low AQ | **< .001** |
| Bin 2, low AQ – Bin 1, high AQ | .992 |
| Bin 2, low AQ – Bin 2, high AQ | .942 |
| Bin 2, low AQ – Bin 3, high AQ | **< .001** |
| Bin 2, low AQ – Bin 4, high AQ | **< .001** |
| Bin 2, low AQ – Bin 5, high AQ | **< .001** |
| Bin 2, low AQ – Bin 6, high AQ | **< .001** |
| Bin 3, low AQ – Bin 4, low AQ | **< .001** |
| Bin 3, low AQ – Bin 5, low AQ | **< .001** |
| Bin 3, low AQ – Bin 6, low AQ | **< .001** |
| Bin 3, low AQ – Bin 1, high AQ | **< .001** |
| Bin 3, low AQ – Bin 2, high AQ | .697 |
| Bin 3, low AQ – Bin 3, high AQ | 1.000 |
| Bin 3, low AQ – Bin 4, high AQ | **< .001** |
| Bin 3, low AQ – Bin 5, high AQ | .341 |
| Bin 3, low AQ – Bin 6, high AQ | .074 |
| Bin 4, low AQ – Bin 5, low AQ | .995 |
| Bin 4, low AQ – Bin 6, low AQ | 1.000 |
| Bin 4, low AQ – Bin 1, high AQ | **< .001** |
| Bin 4, low AQ – Bin 2, high AQ | **< .001** |
| Bin 4, low AQ – Bin 3, high AQ | **< .001** |
| Bin 4, low AQ – Bin 4, high AQ | 1.000 |
| Bin 4, low AQ – Bin 5, high AQ | **.008** |
| Bin 4, low AQ – Bin 6, high AQ | .055 |
| Bin 5, low AQ – Bin 6, low AQ | 1.000 |
| Bin 5, low AQ – Bin 1, high AQ | **< .001** |
| Bin 5, low AQ – Bin 2, high AQ | **< .001** |
| Bin 5, low AQ – Bin 3, high AQ | **.008** |
| Bin 5, low AQ – Bin 4, high AQ | .953 |
| Bin 5, low AQ – Bin 5, high AQ | .917 |
| Bin 5, low AQ – Bin 6, high AQ | .999 |
| Bin 6, low AQ – Bin 1, high AQ | **< .001** |
| Bin 6, low AQ – Bin 2, high AQ | **< .001** |
| Bin 6, low AQ – Bin 3, high AQ | **< .001** |
| Bin 6, low AQ – Bin 4, high AQ | 1.000 |
| Bin 6, low AQ – Bin 5, high AQ | .082 |
| Bin 6, low AQ – Bin 6, high AQ | .365 |
| Bin 1, high AQ – Bin 2, high AQ | **.008** |
| Bin 1, high AQ – Bin 3, high AQ | **< .001** |
| Bin 1, high AQ – Bin 4, high AQ | **< .001** |
| Bin 1, high AQ – Bin 5, high AQ | **< .001** |
| Bin 1, high AQ – Bin 6, high AQ | **< .001** |
| Bin 2, high AQ – Bin 3, high AQ | .053 |
| Bin 2, high AQ – Bin 4, high AQ | **< .001** |
| Bin 2, high AQ – Bin 5, high AQ | **< .001** |
| Bin 2, high AQ – Bin 6, high AQ | **< .001** |
| Bin 3, high AQ – Bin 4, high AQ | **< .001** |
| Bin 3, high AQ – Bin 5, high AQ | .996 |
| Bin 3, high AQ – Bin 6, high AQ | .789 |
| Bin 4, high AQ – Bin 5, high AQ | **.003** |
| Bin 4, high AQ – Bin 6, high AQ | **.023** |
| Bin 5, high AQ – Bin 6, high AQ | 1.000 |

Please note that AQ scores were entered in the analyses as continuous variables, but here, for interpretability, we compute estimates for scores 1 SD below and above the mean.**Supplementary Table S8.** Results of the linear mixed model predicting Speed/accuracy trade-off throughout the task.

| **Prediction of Speed/Accuracy Trade-off Score** | | | | | |
| --- | --- | --- | --- | --- | --- |
| **Fixed Effects** | ***β estimates*** | ***95% CI*** | ***t*** | ***df*** | ***p*** |
| **(Intercept)** | 1.108 | 0.970 – 1.247 | 15.711 | 1764.000 | **< .001** |
| AQ | -0.019 | -0.044 – 0.005 | -1.546 | 1764.000 | .122 |
| **Bin [2]** | -0.508 | -0.704 – -0.312 | -5.092 | 1764.000 | **< .001** |
| **Bin [3]** | -1.055 | -1.250 – -0.859 | -10.574 | 1764.000 | **< .001** |
| **Bin [4]** | -1.932 | -2.128 – -1.737 | -19.371 | 1764.000 | **< .001** |
| **Bin [5]** | -1.519 | -1.715 – -1.323 | -15.226 | 1764.000 | **< .001** |
| **Bin [6]** | -1.635 | -1.831 – -1.440 | -16.394 | 1764.000 | **< .001** |
| AQ × Bin [2] | -0.006 | -0.041 – 0.029 | -0.343 | 1764.000 | .731 |
| AQ × Bin [3] | 0.007 | -0.028 – 0.042 | 0.400 | 1764.000 | .689 |
| AQ × Bin [4] | 0.016 | -0.018 – 0.051 | 0.926 | 1764.000 | .355 |
| **AQ × Bin [5]** | 0.045 | 0.011 – 0.080 | 2.570 | 1764.000 | **.010** |
| **AQ × Bin [6]** | 0.053 | 0.018 – 0.088 | 3.008 | 1764.000 | **.003** |
| **Random Effects** | | | | | |
| σ^2^ | 1.473 | | | | |
| τ_00_ _id_ | 0.000 | | | | |
| N _id_ | 296 | | | | |
| Observations | 1776 | | | | |
| Marginal R^2^ / Conditional R^2^ | 0.242 / NA | | | | |

The table shows regression coefficients of fixed effects and summary information of random effects. The marginal R^2^ considers only the variance of fixed effects, while the conditional R^2^ takes both fixed and random effects into account. Degrees of freedom are based on Satterthwaite’s approximation. Statistically significant terms are highlighted in bold. Terms in brackets indicate the level of factor that is contrasted against the reference level, which is Bin 1 for the Bin factor.
